# Supplementary material for: Does pre-existing morbidity influences risks and benefits of total hip replacement for osteoarthritis: a prospective study of 6682 patients from linked national datasets in England
Source: BMJ Open. 2021 Sep 22;11(9):e046712. doi: 10.1136/bmjopen-2020-046712 (PMC8461685; doi:10.1136/bmjopen-2020-046712)
Supplement: Supplementary data [file bmjopen-2020-046712supp002.pdf]

Appendix: Interpretation of Oxford Hip Scores and EQ-5D in patients receiving a hip arthroplasty

The OHS is a 12-item score with 4 levels, thus scores vary between 0-48, with higher scores being the best outcome. The clinically meaningful difference is considered to be an increase of +11 points and a change of +5 is considered statistically meaningful (ie greater than background 'noise')

Beard DJ, Harris K, Dawson J, *et al.* Meaningful changes for the Oxford hip and knee scores after joint replacement surgery. *J Clin Epidemiol* 2015; **68**: 73–9.

The EQ-5D has been very widely studied in several disease areas. There have been several studies looking at what is a clinically meaningful difference and a meta-analysis yielded an average difference of 0.18

Coretti S, Ruggeri M, McNamee P. The minimum clinically important difference for EQ-5D index: A critical review. Expert Rev. Pharmacoeconomics Outcomes Res. 2014; **14**: 221–33
