# Supplementary material for: Does pre-existing morbidity influences risks and benefits of total hip replacement for osteoarthritis: a prospective study of 6682 patients from linked national datasets in England
Source: BMJ Open. 2021 Sep 22;11(9):e046712. doi: 10.1136/bmjopen-2020-046712 (PMC8461685; doi:10.1136/bmjopen-2020-046712)
Supplement: Supplementary data [file bmjopen-2020-046712supp001.pdf]

**Supplementary Table 1: Baseline data on measures of pre-operative health status (multimorbidity, frailty and morbidity burden) in patients who underwent primary THR in HES APC during the date period in which PROMs were collected (N=2992)**

| | PROMs data | No PROMs data |
|---|---|---|
| **Charlson comorbidity index** | | |
| 0 | 1,013 (68.7) | 931 (61.4) |
| 1 | 123 (8.3) | 152 (10.0) |
| 2 | 205 (13.9) | 254 (16.7) |
| 3+ | 134 (9.1) | 180 (11.9) |
| **Electronic frailty index (eFI)** | | |
| 0-4 | 831 (56.3) | 752 (49.6) |
| 5-8 | 544 (36.9) | 593 (39.1) |
| 9-12 | 93 (6.3) | 151 (10.0) |
| 13+ | 7 (0.5) | 21 (1.4) |
| **No. comorbid diseases** | | |
| 0 | 378 (25.6) | 320 (21.1) |
| 1 | 497 (33.7) | 464 (30.6) |
| 2 | 346 (23.5) | 364 (24.0) |
| 3+ | 254 (17.2) | 369 (24.3) |
| **No. medications prescribed** | | |
| 0-4 | 428 (29.0) | 400 (26.4) |
| 5-7 | 401 (27.2) | 375 (24.7) |
| 8-12 | 429 (29.1) | 500 (33.0) |
| 13+ | 217 (14.7) | 242 (16.0) |
| **No. contacts with primary care** | | |
| 0-7 | 471 (31.9) | 464 (30.6) |
| 8-11 | 335 (22.7) | 358 (23.6) |
| 12-17 | 384 (26.0) | 334 (22.0) |
| 18+ | 285 (19.3) | 361 (23.8) |
| **Total** | 1475 | 1517 |

**Supplementary Table 2**

**Influence of pre-operative health status on risk of hospital readmission within 90 days**

| | No. patients | Events | % Events | Unadjusted OR (95% CI) | Adjusted OR* (95% CI) |
|---|---|---|---|---|---|
| Charlson Comorbidity Index | | | | | |
| 0 | 4490 | 477 | 10.6 | Ref | Ref |
| 1 | 682 | 82 | 12.0 | 1.15 (0.90, 1.48) | 1.09 (0.85, 1.40) |
| 2 | 880 | 131 | 14.9 | 1.47 (1.19, 1.81) | 1.41 (1.14, 1.74) |
| 3+ | 630 | 96 | 15.2 | 1.51 (1.19, 1.92) | 1.45 (1.14, 1.84) |
| Count of chronic diseases | | | | | |
| 0 | 1901 | 192 | 10.1 | Ref | Ref |
| 1 | 2286 | 232 | 10.2 | 1.01 (0.82, 1.23) | 1.01 (0.82, 1.23) |
| 2 | 1430 | 205 | 14.3 | 1.49 (1.21, 1.84) | 1.47 (1.19, 1.83) |
| 3+ | 1065 | 157 | 14.7 | 1.54 (1.23, 1.93) | 1.53 (1.21, 1.94) |
| Count of medications prescribed | | | | | |
| 0-4 | 1963 | 180 | 9.2 | Ref | Ref |
| 5-7 | 1832 | 201 | 11.0 | 1.22 (0.99, 1.51) | 1.22 (0.99, 1.51) |
| 8-12 | 1992 | 257 | 12.9 | 1.47 (1.20, 1.80) | 1.45 (1.18, 1.78) |
| 13+ | 895 | 148 | 16.5 | 1.96 (1.55, 2.48) | 1.96 (1.54, 2.49) |
| Count of contacts with primary care | | | | | |
| 0-7 | 2167 | 204 | 9.4 | Ref | Ref |
| 8-11 | 1598 | 172 | 10.8 | 1.16 (0.94, 1.44) | 1.15 (0.93, 1.43) |
| 12-17 | 1528 | 192 | 12.6 | 1.38 (1.12, 1.70) | 1.37 (1.11, 1.69) |
| 18+ | 1389 | 218 | 15.7 | 1.79 (1.46, 2.20) | 1.75 (1.42, 2.15) |
| Electronic Frailty Index | | | | | |
| 0-4 | 4184 | 432 | 10.3 | Ref | Ref |
| 5-8 | 2064 | 270 | 13.1 | 1.31 (1.11, 1.54) | 1.30 (1.10, 1.55) |
| 9-12 | 393 | 76 | 19.3 | 2.08 (1.59, 2.73) | 2.01 (1.51, 2.67) |
| 13+ | 41 | 8 | 19.5 | 2.11 (0.97, 4.59) | 2.15 (0.97, 4.73) |
| Total | 6682 | 786 | 11.8 | | |

*The model was adjusted for age, sex, region of the residence, and year of surgery.

**Supplementary Table 3:**

**Influence of pre-operative health status on length of stay in hospital**

| | Total at risk | Median length of stay (IQR) |
|---|---|---|
| Charlson Comorbidity Index | | |
| 0 | 4490 | 6 (4, 9) |
| 1 | 682 | 6 (5, 9) |
| 2 | 880 | 6 (4, 9) |
| 3+ | 630 | 7 (5, 9) |
| Count of chronic diseases | | |
| 0 | 1901 | 6 (4, 8) |
| 1 | 2286 | 6 (4, 9) |
| 2 | 1430 | 6 (4, 9) |
| 3+ | 1065 | 7 (5, 10) |
| Count of medications prescribed | | |
| 0-4 | 1963 | 6 (4, 8) |
| 5-7 | 1832 | 6 (4, 9) |
| 8-12 | 1992 | 7 (5, 9) |
| 13+ | 895 | 7 (5, 10) |
| Count of contacts with primary care | | |
| 0-7 | 2167 | 6 (4, 8) |
| 8-11 | 1598 | 6 (4, 9) |
| 12-17 | 1528 | 6 (4, 9) |
| 18+ | 1389 | 7 (5, 10) |
| Electronic Frailty Index | | |
| 0-4 | 4184 | 6 (4, 9) |
| 5-8 | 2064 | 6 (4, 9) |
| 9-12 | 393 | 8 (5, 11) |
| 13+ | 41 | 8 (7, 13) |
| Total | 6682 | 6 (4, 9) |

| ,
