# Supplementary material for: Does pre-existing morbidity influences risks and benefits of total hip replacement for osteoarthritis: a prospective study of 6682 patients from linked national datasets in England
Source: BMJ Open. 2021 Sep 22;11(9):e046712. doi: 10.1136/bmjopen-2020-046712 (PMC8461685; doi:10.1136/bmjopen-2020-046712)
Supplement: Author's manuscript [file bmjopen-2020-046712.draft_revisions.pdf]

BMJ Open is committed to open peer review. As part of this commitment we make the peer review history of every article we publish publicly available.

When an article is published we post the peer reviewers' comments and the authors' responses online. We also post the versions of the paper that were used during peer review. These are the versions that the peer review comments apply to.

The versions of the paper that follow are the versions that were submitted during the peer review process. They are not the versions of record or the final published versions. They should not be cited or distributed as the published version of this manuscript.

BMJ Open is an open access journal and the full, final, typeset and author-corrected version of record of the manuscript is available on our site with no access controls, subscription charges or pay-per-view fees (http://bmjopen.bmj.com).

If you have any questions on BMJ Open's open peer review process please email info.bmjopen@bmj.com

BMJ Open

Does pre-existing morbidity influence risks and benefits of total hip replacement for osteoarthritis: a prospective study of 6682 patients from linked national datasets in England?

Journal:	*BMJ Open*
Manuscript ID	bmjopen-2020-046712
Article Type:	Original research
Date Submitted by the Author:	11-Nov-2020
Complete List of Authors:	Ferguson, Rory; University of Oxford Nuffield Department of Orthopaedics Rheumatology and Musculoskeletal Sciences, Botnar Institute<br>Prieto-Alhambra, Daniel; University of Oxford, Centre for Statistics in Medicine, NDORMS<br>Peat, George; Keele University, Research Institute for Primary Care & Health Sciences<br>Delmestri, Antonella; University of Oxford, Nuffield Department of Orthopaedics, Rheumatology, and Musculoskeletal Sciences<br>Jordan, Kelvin; Keele University, School of Primary, Community and Social Care<br>Strauss, Vicky; University of Oxford, Centre for Statistics in Medicine, Ndorms<br>Valderas, Jose; University of Exeter Medical School,<br>Walker, Christine; Keele University, Research User Group, Arthritis Research UK Primary Care Centre, Research Institute of Primary Care and Health Sciences<br>Yu, Dahai; Keele University, Research Institute for Primary Care & Health Sciences<br>Glyn-Jones, Sion; University of Oxford, NDORMS<br>Silman, Alan; University of Oxford Nuffield Department of Orthopaedics Rheumatology and Musculoskeletal Sciences, Botnar Institute
Keywords:	Hip < ORTHOPAEDIC & TRAUMA SURGERY, GERIATRIC MEDICINE, SURGERY

SCHOLARONE™
Manuscripts

I, the Submitting Author has the right to grant and does grant on behalf of all authors of the Work (as defined in the below author licence), an exclusive licence and/or a non-exclusive licence for contributions from authors who are: i) UK Crown employees; ii) where BMJ has agreed a CC-BY licence shall apply, and/or iii) in accordance with the terms applicable for US Federal Government officers or employees acting as part of their official duties; on a worldwide, perpetual, irrevocable, royalty-free basis to BMJ Publishing Group Ltd ("BMJ") its licensees and where the relevant Journal is co-owned by BMJ to the co-owners of the Journal, to publish the Work in this journal and any other BMJ products and to exploit all rights, as set out in our licence.

The Submitting Author accepts and understands that any supply made under these terms is made by BMJ to the Submitting Author unless you are acting as an employee on behalf of your employer or a postgraduate student of an affiliated institution which is paying any applicable article publishing charge ("APC") for Open Access articles. Where the Submitting Author wishes to make the Work available on an Open Access basis (and intends to pay the relevant APC), the terms of reuse of such Open Access shall be governed by a Creative Commons licence – details of these licences and which Creative Commons licence will apply to this Work are set out in our licence referred to above.

Other than as permitted in any relevant BMJ Author's Self Archiving Policies, I confirm this Work has not been accepted for publication elsewhere, is not being considered for publication elsewhere and does not duplicate material already published. I confirm all authors consent to publication of this Work and authorise the granting of this licence.

For peer review only

Does pre-existing morbidity influence risks and benefits of total hip replacement for osteoarthritis: a prospective study of 6682 patients from linked national datasets in England

RJ Ferguson [1]
D Prieto-Alhambra [1]
G Peat [2]
A Delmestri [1]
KP Jordan [2]
V Strauss [1]
JM Valderas [3]
C Walker [2]
D Yu[2]
S Glyn-Jones [1]
AJ Silman[1]

[1]Nuffield Department of Orthopaedics, Rheumatology and Musculoskeletal Sciences, University of Oxford, Oxford, UK.
[2]Primary Care and Health Sciences Department, Keele University, Keele, UK.
[3]University of Exeter Medical School, Exeter, UK.
[4]Oxford University Hospital NHS Foundation Trust, Oxford, UK.

Correspondence to Professor Silman
Nuffield Department of Orthopaedics, Rheumatology and Musculoskeletal Sciences, University of Oxford
Botnar Research Centre, Windmill Road
Headington, Oxford, OX3 7LD.
alan.silman@ndorms.ox.ac.uk

Acknowledgements

We acknowledge Professor Andy Judge for advice on statistical analysis at the earliest stages of this project . We are also grateful to Dr Andrew Clegg, and Stephen Pye for guidance in the use of the electronic frailty index. Dr John Griffiths and Professor Nigel Arden, provided very helpful advice at the design stage on the challenges of anaesthetic assessment and on studying osteoarthritis from an epidemiological perspective.

Word count abstract, main paper 3424

Key words: Hip osteoarthritis, arthroplasty, multimorbidity, mortality, outcome, quality of life.

For peer review only

Abstract

Objective

To evaluate how pre-existing health status, using multiple approaches, influences the relative risks of, and quality of life benefits from, THA.

Design

Longitudinal record linkage study

Setting

UK national sample of linked primary and secondary care registers.

Participants

All 6682 patients, between 1995 and 2014, with hip osteoarthritis identified from the national primary care register who had a THA. Data from their primary care record was extracted to score 5 different constructs: Charlson Comorbidity Index, Electronic Frailty Index (eFI) and counts of comorbidity disorders (from list of 17), prescribed medications and number of primary care visits

Outcome measures

Linked secondary care register provided data on mortality and major medical complications within 90 days of surgery. Linkage to the national Patients Reported Outcome Measures (PROMS) database permitted assessment of change in hip specific and general improvement in quality of life using the Oxford Hip Score (OHS) and EQ5D respectively

Results

Peri-operative complication rate was 3.2% and mortality was 0.9%, both of which increased with worse pre-operative health status but with only modest increases in the highest risk strata: all adjusted odd ratios under 2.0 exception being eFI: most frail OR=4.3; 95%CI [0.95-19.7]). By contrast, the benefits were not reduced in those with more adverse pre-operative health: PROMS improvement with either OHS or EQ5D, was similar across morbidity strata

Conclusions

Independent of pre-operative morbidity, THA leads to similar but substantial improvements in quality of life. These are offset by an increase in medical complications in the small subgroup of patients with high morbidity. For most elderly people, their other health disorders should not be a barrier for THA.

(267 words)

Strengths and limitation

- National large sample of older patients recorded in primary care with newly reported osteoarthritis of the hip and who had received a hip replacement
- Multi-dimensional approach to assessing their concurrent morbidity and health status prior to surgery from the primary care record
- Linkage to their surgical outcomes both hazards - mortality and significant post-operative complications and the benefits - improvement in quality of life
- Such study using routine national datasets have advantage of size, access to complete health record and linkage to both data hazards and benefits from surgery
- Relying on routine data has the challenges of completeness of recording and accuracy of the data items extracted

For peer review only

Background

Total hip arthroplasty (THA) results in a substantial improvement in quality of life [1,2]. Most recipients are older adults: the median age in many countries approaching 70 years.[1]

With increasing age, patients are increasingly likely to develop multiple chronic health disorders (multimorbidity) with many patients described as frail. [3–7] Such disorders increase the hazards from surgery[8-10] and may also limit the benefits in quality of life.[11-13] There is in practice an inevitable selection process prior to surgical acceptance of older adults with multimorbidity, though there is no specific guidance on how these factors should influence surgical decision making, either in UK [14] or USA [15]:

There are challenges in identifying a valid approach to measure the cumulative severity of all chronic health disorders.[16] Simple counts of the presence of specific chronic diseases, or weighted instruments (eg Charlson score), typically focus on disorders associated with the hazards but exclude those that might impact on quality of life.

Our goal was to identify the impact that multimorbidity and frailty on the risks and benefits of THA. Our specific objectives were to assess, using a number of different approaches to scoring, how multimorbidity influenced the risks and benefits of elective total hip replacement for osteoarthritis. The former outcome was assessed by post-operative complications, length of stay and hospital readmission; and the latter assessed by patient reported outcome scores (PROMS) covering pain, function and quality of life. To maximise the external validity, the study took advantage of the availability of English national datasets linking primary and secondary care

Methods

Summary of design

In a prospective design using a national database, patients with a newly recorded diagnosis of hip osteoarthritis were identified from their primary care record. Linkage to the national secondary care database identified those individuals who had a THA, who became the subjects of the study. The primary care record was interrogated to provide information on other health disorders and treatments were used to derive measures of pre-existing multimorbidity and health status at the time of surgery. This secondary care database also provided data on peri-operative death and major complications as well as scores of patient outcome. The influence of the scores from these measures on mortality, in-patient complications and readmission within 90 days and PROMS at 6 months were then calculated.

Data sources

We used the UK Clinical Practice Research Datalink (CPRD) which contains the primary care electronic medical records of approximately 4.4 million active patients and representative of

For peer review only - http://bmjopen.bmj.com/site/about/guidelines.xhtml

the wider UK population.[17] Data are stored with Read codes for diseases that are cross-referenced to the International Classification of Diseases (ICD-10).

The Hospital Episode Statistics dataset (HES) contains all patient attendances at NHS hospitals in England, and covers episodes of care. HES records patient demographic data, diagnoses and procedures and is linked to national death certificate data. HES has also been linked since 2009 to the PROMs database gathering outcome data NHS-funded THA both pre-operatively and at 6 months post-operatively.[18]

Study Population

All patients in the CPRD with a READ code for hip osteoarthritis from January 1 1995, until March 31, 2014, and aged 65 years or over at the time of diagnosis, were identified. The validation of those Read codes for hip osteoarthritis are described elsewhere.[19] Those patients who underwent elective primary THA, identified from HES were included in the analysis. Operations were identified from the Classification of Surgical Operations and Procedures (OPCS-4) codes.

The following measures of multimorbidity prior to the date of surgery were extracted from the accumulated primary care record. These were: (i) Charlson Comorbidity Index (CCI)- developed to predict 1-year mortality based on the presence of specified chronic diseases.[20], (ii) count of the 17 chronic diseases listed in the NHS Quality Outcomes Framework (QOF).[21] (iii) electronic Frailty Index (eFI), a score based on the cumulative deficit model of frailty and validated against mortality, hospitalisation and nursing home admission.[22] We also used 2 measures of the burden of care associated with the presence of chronic conditions. Thus within the 12 months prior to surgery we extracted the numbers of (i) different prescribed medications and (ii) primary care contacts for any reason.

Outcomes

All subjects had the following outcomes, in addition to death, extracted from the linked HES data within 90 days of THA: venous thromboembolism, myocardial infarction, stroke, anaemia, lower respiratory tract infection, urinary tract infection, wound infection. Also extracted were length of stay following surgery and readmission after discharge within 90 days. The national PROMs database provided data since 2009 on (i) Oxford Hip Score as a measure of hip function and (ii) EQ-5D Index as a measure of quality of life at 6 months post-operatively on all patients receiving a THA. Although all patients are invited to complete the PROMs questionnaires pre-operatively and at 6 months post-operatively, completion rates vary.[20]

Statistical analysis

The scores for each of the five measures of multimorbidity were split into 4 categories. The eFI has 4 categories defined by the developers: fit (0-4), mild frailty (5-8), moderate frailty (9-

For peer review only

For peer review only - http://bmjopen.bmj.com/site/about/guidelines.xhtml

12), and severe frailty (13+). The actual CCI scores were used to split into categories of score of 0, 1, 2, and 3+. Based on the distribution of the data the counts of number of chronic diseases were also split in categories of score of 0, 1, 2, and 3+. The categories for the number of medications prescribed and of primary care visits were derived from the observed distributions of the actual data, aiming for equal sized quarters: with medication counts divided into scores of 0-4, 5-7, 8-12, and 13+ and number of primary care visits into 0-7, 8-11, 12-17 and 18+. The lowest category for each score was used as the referent category in the analysis.

Logistic regression methods were used to calculate the unadjusted odds of a complication or readmission to hospital by multimorbidity category. Poisson regression methods were used to calculate the unadjusted difference in length of stay by multimorbidity category. Linear regression methods were used to calculate the difference in post-operative OHS and EQ-5D score, adjusted for pre-operative score, by multimorbidity category. All models were then adjusted for age at surgery, sex, region of the UK, and calendar year of total hip replacement.

Patient and Public Involvement

This research was funded by the NIHR Research for Patient Benefit scheme which has an absolute requirement that there is patient and public involvement in all relevant stages of the research. The research question had been originally raised in a "Priority Setting Partnership for Priorities for Research in Hip and Knee Arthroplasty" and this was followed by a survey of members of the Keele PPI panel: Research Users Group (RUG). We then tested the suggested questions with the group and received very positive feedback. One of the RUG members (Author CW), herself a patient with direct experience of the target of the research, then became an active member of the research team, participated in all the meetings and advised on the questions that should be asked during the analysis stage. As part of the dissemination phase, Keele PPI group organised a round table event attended by the lead authors where the results were discussed and guidance given on how those members of the public who were present wished to see disseminated

Results

Baseline data

The demographic characteristics of the study participants are shown in Table 1. There were 6682 patients with a code for hip osteoarthritis on the primary care record who after linkage to the HES data, had had a THA within the follow up period from April 1997 to March 2014. The mean age at surgery was 76 years (SD 6).

Pre-operative scores for the four multimorbidity measures derived are shown in Table 2. Only 20% had a CCI above 1 perhaps reflecting the selection of patients for surgery. Based on the eFI classification, 34% were classified as 'mildly frail' and 6.5% 'moderately frail'.

For peer review only - http://bmjopen.bmj.com/site/about/guidelines.xhtml

Around 1 in 6 had 3 or more chronic disorders recorded. Over 40% had been prescribed at least 8 medications, and a similar proportion had more than 12 primary care visits, in the previous 12 months

Outcomes

Data on complications, length of stay and readmission to hospital were available on all patients (as linkage to HES was a criterion for study entry). As mentioned above, data on PROMs was only available on a subset of patients who completed the questionnaires (Figure). In total, pre- and post-operative OHS was available on 1402 patients, and pre- and post-operative EQ-5D Index was available on 1285 patients. As a test of bias, we compared the multimorbidity measures between those with and without PROMS (Supplementary Table 1) which whilst showing no substantive shifts, across all the measures, those who did not provided PROMS were more likely to have worse multimorbidity and also reside in more socio economically deprived areas.

Association between multimorbidity measures and adverse outcomes

In all, 216 (3.2%) patients suffered a post-operative complication including thrombo-embolism, myocardial infarction, urine and respiratory infections and wound infection, and 57 (0.9%) patients died, within 90 days of surgery (Table 3). The rate of all-cause readmission to hospital within 90 days of surgery was 11%.

The impact of multimorbidity scores across all these medical complications are shown in Table 4. The overall adverse event rate was modestly increased with increasing levels of multimorbidity relative to the lowest scoring group. These results were similar result across the different approaches to assessing multimorbidity. Given the large numbers in the study the confidence intervals were relatively narrow. The actual increases in risks were similarly modest, both before and after age adjustment. Those in highest frailty group were the most predictive, compared to the other measures of multimorbidity, of a medical complication. We analysed the individual complications separately (data not shown). The numbers with any of the individual complications were modest but there were no substantial difference between the risks of individual complications in their relationship with the multimorbidity scores.

The data on mortality are shown in Table 5. The mortality risk in the least healthy stratum ranged from 0.7% for primary care contacts to 2.3% for those with an eFI of 9 or more. There was an inconsistent association between death and Charlson score but the number of events in those greater than a score of 2 were too small for useful analysis. Interestingly the category of count of medications prescribed did not affect the mortality rate. The measure with the greatest predictive power of death was the eFI. After adjustment for age and gender, those in the two highest frailty groups had a 2.5 fold increased mortality risk.

For peer review only

The impact of multimorbidity scores on length of stay were also analysed. Detailed data are not shown but in brief for all the multimorbidity measures, apart from the eFI, there was no impact on length of stay. The median length of stays in the lowest and highest categories of multimorbidity were: 6 and 7 days for Charlson score, 6 and 8 days for eFI score, 6 and 7 days for the count of chronic diseases, and 6 and 7 days for the count of medications prescribed.

Association between multimorbidity measures and quality of life

Across all patients, there was an overall marked improvement in both the specific (OHS) and the generic (EQ5D) patient outcomes. The summary outcome data are shown in Table 6, confirming substantial improvement at 6 months in both the OHS and EQ-5D across of all the population studied. The impact of multimorbidity scores on both the pre-operative and post -operative OHS are shown in Table 7. For each of our measures of multimorbidity, the pre-operative OHS showed was lower with increasing score. What was of note was the similarity in the relative improvement in the post -operative OHS seen across the board for all measures of multimorbidity. Typically mean pre-operative scores in the mid to late teens reached scores in the high 30's and 40's: the highest score achievable is 48. Thus, surgery led to a marked improvement, not attenuated by the severity of the pre-operative general health.

A similar analysis was undertaken on EQ5D (Table 8). As expected, those with a higher multimorbidity score, pre-operatively had a lower EQ5D, reflecting the impact of the other multimorbidity disorders on their overall quality of life. However, the 6 month incremental improvement in EQ5D was almost identical across all categories of multimorbidity, independent of the method of multimorbidity scoring. The actual EQ5D scores were moderately lower in the highest categories of multimorbidity but the relative improvement was the same. Regression analyses, both unadjusted and adjusted for age and gender showed that the post-operative scores, adjusted for the pre-operative scores, were not importantly different for any of the multimorbidity scores compared with the lowest score in each grading system.

Discussion

This is the first study to comprehensively assess the impact of pre-operative multimorbidity, on the risks and benefits of THA. We have demonstrated modest increases in death and clinically significant medical event rates. The other important result was that THA is associated with a substantial improvement in quality of life, independent of level of pre-existing multimorbidity.

There are some limitations of these data. Firstly, we have not considered the impact of different types of surgery and anaesthesia together with other prophylactic interventions to

For peer review only - http://bmjopen.bmj.com/site/about/guidelines.xhtml

reduce the hazards from surgery in this group of patients. For example, the routine use of the use of thromboprophylaxis should reduce the risk of thromboembolism even in those with the highest multimorbidity. Such interventions would not, though, explain the observation that improvement in patient outcome is not materially affected by pre-exiting morbidity.

Secondly, inevitably in studies of this type, there is a reliance on the completeness, and quality diagnostic accuracy in the data sets available. We relied on the quality of the CPRD to provide information on the diagnosis of osteoarthritis of the hip and of the multimorbidity scores. As regards the former, a validation study using radiological and clinical information from a subsample of this cohort, described elsewhere, confirmed the diagnosis of hip osteoarthritis in 80 per cent.[19] As regards the latter there are many reports on the validity of the national datasets used for deriving multimorbidity information. [23-25]

There is also a similar reliance on the quality of the outcome data from HES. Although data from HES is widely accepted as being sufficiently valid [26] we were concerned that this data source captured a high proportion of these relevant outcome events with minimal misclassification. As a test of the quality of the outcomes data, we therefore compared our results on post-operative hazard rates with other published data to determine if such rates were broadly similar. There are limitations in such comparisons, but this analysis suggested that the observed rates of clinically significant events in the population studied did accord with those published from other countries. For example, our VTE incidence was 2.1%, published population data suggests a range of 0.7-3.9%.[26–29] Similar comparisons for MI were: present study 0.4%, literature 0.4-0.6%[30,31]; wound infection present study 1.7%, literature 2.2-2.9%.[29,32]

We specifically studied multiple ways of measuring pre-existing morbidity and its impact on health, given there is no single 'best buy' and all such scores are measures of an underlying, but hard-to-measure, construct. This resulted in multiple estimates of effect. We were not attempting to prove statistical significance by finding one or more measure that was useful. More, by using multiple options, we covered as far as possible the different suggested approaches to ascertaining multimorbidity. These measures, whilst overlapping in the patients classified, were far from being concordant, but this is not surprising as they were measuring different constructs. Despite such differences, none of the scoring systems had substantial effects on outcome.

There are other variables that might be confounders which have not been addressed in the analyses presented which include socio-economic status, body mass index (BMI), and cigarette smoking. The former can be indirectly derived from the area served by the primary care provider using the Index of Multiple Deprivation (IMD). Smoking and BMI data in the primary care record are not routinely collected and the former, in particular, might be biased

For peer review only - http://bmjopen.bmj.com/site/about/guidelines.xhtml

towards to being selectively collected in those who smoked. Indeed only 8% of records mentioned smoking as being present in the year preceding osteoarthritis mention. The data on BMI and IMD are, however, shown in Table 1. We specifically did not adjust for the possible confounding effects of these variables in part because of the missing data. More importantly, we were concerned that the effect of these variables would most likely be mediated by their association with other comorbid disorders and interpretation of such adjusted results would be difficult.

Finally, the patients studied in this cohort were inevitably those who underwent surgery. Our comparisons therefore were between those in the different morbidity categories for whom a decision had been made that surgery was appropriate. We could not address whether, *within each such category*, there were selective differences in surgical decision making that might be associated with the outcomes measured.

This study aimed to investigate the influence of common long-term disorders in the population and their influence on the risks and benefits from hip surgery. It is accepted that those at greatest risk from anaesthesia and major surgery should be identified, especially for an elective procedure such as THA. By contrast, there are very little data on how the co-existing health problems affect the clinical benefits. Indeed, we have shown in a recent analysis that those THA patients in the highest American Society of Anesthesiologists (ASA)[33] grade (III and above) are at increased risk of revision surgery[34], a result that is not unexpected. ASA grades whilst routinely collected for example in national joint registers, are not extractable from primary care data systems. The underlying concern behind undertaking the current study was that, especially in older individuals, the lack of an evidence base weighing up the risks and benefits from surgery, leads to patients, their families and health care professionals, accepting that surgery should be avoided. The current study suggests that the increased risks are modest and the benefits similar.

As part of the study design, we have been able to examine a number of different approaches to assessing multimorbidity in primary care patients. Indeed, that setting with the complete medical record provides the only practical one for scoring patients in clinical practice. The eFI, a recently developed tool for assessing frailty from the primary care record[22], is achieving widespread usage in the UK and elsewhere for classifying frailty in elderly patients. High eFI scores are associated with substantial increased risks of mortality, unplanned hospital admission and the need for long term care [22]. It is thus interesting that within the current dataset the differences in eFI class were, if anything more predictive of outcomes than the other approaches. It should be noted though that the proportion of the population selected for surgery in the highest eFI category was small. Further, even a high eFI class on our data, should not, of itself, be a barrier to surgery.

For peer review only - http://bmjopen.bmj.com/site/about/guidelines.xhtml

We conclude that in older patients requiring a hip replacement, unless they are in the small group whose anaesthetic risk precludes safe surgery, there is no evidence that their other long-term disorders should be a barrier to this life changing elective procedure.

For peer review only

For peer review only - http://bmjopen.bmj.com/site/about/guidelines.xhtml

Contributorship Statement: The study was conceived by AJS with SG-J and DP-A and detailed protocol produced by these authors with input from GP, KPJ, JMV and DY. AD was responsible for extracting, preparing and ensuring the quality of the datasets for analysis; analysis was undertaken by RJS supported by VS, DP-A and AJS. CW was the PPI representative in the research group and contributed to the design, approach to analysis and the interpretation of the results from a PPI perspective. All authors contributed to the discussion of the results. The manuscript was prepared by AJS and RJF and reviewed by all authors

Competing interests:

The authors declare that they have no competing interests to report

Funding

This report is independent research funded by the National Institute for Health Research (Research for Patient Benefit programme, PB-PG-0815-20024). The views expressed in this publication are those of the authors and not necessarily those of the NIHR or the Department of Health and Social Care

This study is based in part on data from the Clinical Practice Research Datalink (CPRD) obtained under licence from the UK Medicines and Healthcare products Regulatory Agency. The data is provided by patients and collected by the NHS as part of their care and support. The interpretation and conclusions contained in this study are those of the author/s alone
HES Data/ONS Data shown are Copyright © 2016 are re-used with the permission of The Health & Social Care Information Centre. All rights reserved.
The OPCS Classification of Interventions and Procedures, codes, terms and text is Crown copyright (2016) published by Health and Social Care Information Centre, also known as NHS Digital and licensed under the Open Government Licence available at www.nationalarchives.gov.uk/doc/open-government-licence/open-government-licence.htm.

Ethical Approval

No ethical approval was required and anonymised data were provided by as above. No individual patient consent was therefore required

Consent for publication: not applicable

Data sharing statement: The data that support the findings of this study are available from CPRD but restrictions apply to the availability of these data, which were used under license for the current study, and so are not publicly available. The datasets extracted from CPRD and used in the analysis during the current study are available from the corresponding author on reasonable request and consistent with the permission received from CPRD

For peer review only - http://bmjopen.bmj.com/site/about/guidelines.xhtml

References

1 National Joint Registry. National Joint Registry for England, Wales, Northern Ireland and the Isle of Man: 15th Annual Report. 2018.

2 NHS Digital. Provisional Quarterly Patient Reported Outcome Measures (PROMs) in England. 2017. https://digital.nhs.uk/catalogue/PUB23060.

3 Barnett K, Mercer SW, Norbury M, Watt G, Wyke S, Guthrie B. Epidemiology of multimorbidity and implications for health care, research, and medical education: a cross-sectional study. *Lancet* 2012; **380**: 37–43.

4 Violan C, Foguet-Boreu Q, Flores-Mateo G, *et al.* Prevalence, Determinants and Patterns of Multimorbidity in Primary Care: A Systematic Review of Observational Studies. *PLoS One* 2014; **9**: e102149.

5 Abad-Díez JM, Calderón-Larrañaga A, Poncel-Falcó A, *et al.* Age and gender differences in the prevalence and patterns of multimorbidity in the older population. *BMC Geriatr* 2014; **14**: 75.

6 Gale CR, Cooper C, Aihie Sayer A. Prevalence of frailty and disability: findings from the English Longitudinal Study of Ageing. Age and ageing. 2014 Oct 12;44(1):162-5.

7 Vetrano DL, Palmer K, Marengoni A, Marzetti E, Lattanzio F, Roller-Wirnsberger R, Lopez Samaniego L, Rodríguez-Mañas L, Bernabei R, Onder G, Joint Action ADVANTAGE WP4 Group. Frailty and multimorbidity: a systematic review and meta-analysis. The Journals of Gerontology: Series A. 2018 May 3;74(5):659-66.

8 Aynardi M, Jacovides CL, Huang R, Mortazavi SMJ, Parvizi J. Risk Factors for Early Mortality Following Modern Total Hip Arthroplasty. *J Arthroplasty* 2013; **28**: 517–20.

9 Rasouli MR, Restrepo C, Maltenfort MG, Purtill JJ, Parvizi J. Risk Factors for Surgical Site Infection Following Total Joint Arthroplasty. *J Bone Jt Surgery-American Vol* 2014; **96**: e158-1–5.

10 Singh JA, Jensen MR, Harmsen WS, Gabriel SE, Lewallen DG. Cardiac and thromboembolic complications and mortality in patients undergoing total hip and total knee arthroplasty. *Ann Rheum Dis* 2011; **70**: 2082–8.

11 Gordon M, Paulsen A, Overgaard S, Garellick G, Pedersen AB, Rolfson O. Factors influencing health-related quality of life after total hip replacement - a comparison of data from the Swedish and Danish hip arthroplasty registers. *BMC Musculoskelet Disord* 2013; **14**: 316.

12 Greene ME, Rolfson O, Gordon M, Garellick G, Nemes S. Standard Comorbidity Measures Do Not Predict Patient-reported Outcomes 1 Year After Total Hip Arthroplasty. *Clin Orthop Relat Res* 2015; **473**: 3370–9.

13 Loth FL, Giesinger JM, Giesinger K, *et al.* Impact of Comorbidities on Outcome After Total Hip Arthroplasty. *J Arthroplasty* 2017; **32**: 2755–61.

14 National Institue for Health and Clinical Excellence: https://www.nice.org.uk/guidance/ta304/chapter/4-Evidence-and-interpretation

15 American Academy of Orthopedic Surgeons:

For peer review only - http://bmjopen.bmj.com/site/about/guidelines.xhtml

https://www.aaos.org/uploadedFiles/PreProduction/Quality/Guidelines_and_Reviews/OA%20Hip%20CPG_4-22-19.pdf

16 The Academy of Medical Sciences. Multimorbidity: a priority for global health research. 2018 https://acmedsci.ac.uk/file-download/82222577 (accessed April 4, 2019).

17 Charlson ME, Pompei P, Ales KL, MacKenzie CR. A new method of classifying prognostic comorbidity in longitudinal studies: development and validation. *J Chronic Dis* 1987; **40**: 373–83

18 Glassou EN, Pedersen AB, Aalund PK, Mosegaard SB, Hansen TB: Is gain in health-related quality of life after a total hip arthroplasty depended on the comorbidity burden? Acta Orthop 2018 **89** 374-9

19 Herrett E, Gallagher AM, Bhaskaran K, *et al.* Data Resource Profile: Clinical Practice Research Datalink (CPRD). *Int J Epidemiol* 2015; **44**: 827–36.

20 NHS Digital. Patient Reported Outcome Measures (PROMs). https://digital.nhs.uk/data-and-information/data-tools-and-services/data-services/patient-reported-outcome-measures-proms#guidance (accessed June 21, 2019).

21 Ferguson RJ, Prieto-Alhambra D, Walker C, *et al.* Validation of hip osteoarthritis diagnosis recording in the UK Clinical Practice Research Datalink. *Pharmacoepidemiol Drug Saf* 2018; **28**: 187–93.

22. NHS Primary Care Commissioning *QOF implementation business rules: v11*. http://www.primarycarecontracting.nhs.uk/145.php

23 Clegg A, Bates C, Young J, *et al.* Development and validation of an electronic frailty index using routine primary care electronic health record data. *Age Ageing* 2016; **45**: 353–60.

24 Jick SS(1), Kaye JA, Vasilakis-Scaramozza C, *et al*. Validity of the general practice research database. *Pharmacotherapy*. 2003;**23**:686-9.

25 Herrett E, Gallagher AM, Bhaskaran K), *et al.* Data Resource Profile: Clinical Practice Research Datalink (CPRD). *Int J Epidemiol*. 2015;**44**:827-36.

26 Royal College of Surgeons of England, Academy of Medical Royal Colleges. Hospital Episode Statistics and revalidation.2013, RCS England

27 Senay A, Trottier M, Delisle J, *et al.* Incidence of symptomatic venous thromboembolism in 2372 knee and hip replacement patients after discharge: data from a thromboprophylaxis registry in Montreal, Canada. *Vasc Health Risk Manag* 2018; **Volume 14**: 81–9.

28 Lee S, Hwang J-I, Kim Y, Yoon PW, Ahn J, Yoo JJ. Venous Thromboembolism Following Hip and Knee Replacement Arthroplasty in Korea: A Nationwide Study Based on Claims Registry. *J Korean Med Sci* 2016; **31**: 80.

29 Pedersen AB, Sorensen HT, Mehnert F, Overgaard S, Johnsen SP. Risk Factors for Venous Thromboembolism in Patients Undergoing Total Hip Replacement and

For peer review only

Receiving Routine Thromboprophylaxis. *J Bone Jt Surgery-American Vol* 2010; **92**: 2156–64.

30 Wallace G, Judge A, Prieto-Alhambra D, de Vries F, Arden NKNK, Cooper C. The effect of body mass index on the risk of post-operative complications during the 6 months following total hip replacement or total knee replacement surgery. *Osteoarthr Cartil* 2014; **22**: 918–27.

31 Pedersen AB, Mehnert F, Sorensen HT, Emmeluth C, Overgaard S, Johnsen SP. The risk of venous thromboembolism, myocardial infarction, stroke, major bleeding and death in patients undergoing total hip and knee replacement. *Bone Joint J* 2014; **96**-**B**: 479–85.

32 Belmont PJ, Goodman GP, Kusnezov NA, *et al.* Postoperative Myocardial Infarction and Cardiac Arrest Following Primary Total Knee and Hip Arthroplasty: Rates, Risk Factors, and Time of Occurrence. *J Bone Jt Surgery-American Vol* 2014; **96**: 2025–31.

33 Friedman R, Homering M, Holberg G, Berkowitz SD. Allogeneic Blood Transfusions and Postoperative Infections After Total Hip or Knee Arthroplasty. *J Bone Jt Surg* 2014; **96**: 272–8.

34 Doyle JD, Garmon EH. American Society of Anesthesiologists Classification (ASA Class) https://www.ncbi.nlm.nih.gov/books/NBK441940/

35 Ferguson RJ, Silman AJ, Combescure C), *et al.* ASA class is associated with early revision and reoperation after total hip arthroplasty: an analysis of the Geneva and Swedish Hip Arthroplasty Registries. Acta Orthop. 2019;**90**:324-330.

For peer review only

Figure: Flow chart of participants

For peer review only

For peer review only - http://bmjopen.bmj.com/site/about/guidelines.xhtml

Table 1: Demographic characteristics of cohort

**Gender**	N (%)
Female	4,090 (61.2)
**Age**	
65-69	1,294 (19.4)
70-74	1,976 (29.6)
75-79	1,736 (26.0)
80-84	1,095 (16.4)
85-89	465 (7.0)
>90	116 (1.7)
**Body Mass Index (BMI)**	
Underweight (<18.5)	54 (1.0)
Normal: (18.5-24.9)	1,615 (28.8)
Overweight: (25-29.9)	2,444 (43.6)
Obese: (>30)	1,497 (26.7)
Missing	1072
**Index of multiple deprivation**	
1 (Affluent)	1076 (25.5)
2	1718 (25.7)
3	1539 (23.0)
4	1144 (17.1)
5 (Deprived)	573 (8.6)
Missing	2

For peer review only

Table 2: Baseline data on measures of pre-operative health status (multimorbidity, frailty and morbidity burden)

**Charlson comorbidity index**	
0	4,490 (67.2)
1	682 (10.2)
2	880 (13.2)
3+	630 (9.4)
**Electronic frailty index (eFI)**	
0-4	4184 (62.6)
5-8	2,064 (30.9)
9-12	393 (5.9)
13+	41 (0.6)
**No. comorbid diseases**	
0	1,901 (28.5)
1	2,286 (34.2)
2	1,430 (21.4)
3+	1,065 (15.9)
**No. medications prescribed**	
0-4	1,963 (29.4)
5-7	1,832 (27.4)
8-12	1,992 (29.8)
13+	895 (13.4)
**No. contacts with primary care**	
0-7	2,167 (32.4)
8-11	1,598 (23.9)
12-17	1,528 (22.9)
18+	1,389 (20.8)

For peer review only

Table 3: Complications, length of stay, and readmission to hospital within 90 days

	N (%)
Any medical complication*	216 (3.2)
Death	57 (0.9)
Myocardial infarction	20 (0.3)
Venous thrombo-embolism	54 (0.8)
Stroke	8 (0.1)
Anaemia	12 (0.2)
Respiratory tract infection	28 (0.4)
Urinary tract infection	12 (0.2)
Wound infection	37 (0.6)
Length of stay (median, IQR)	6 (4-9)
Readmission to hospital	727 (10.9)

*Any medical complication includes death, myocardial infarction, venous thrombo-embolism, stroke, anaemia, respiratory tract infection, urinary tract infection, and wound infection

For peer review only

Table 4: Association between pre-operative morbidity status and 90-day medical complications

	No. at risk	Events	% Events	Unadjusted OR	Adjusted OR*
Charlson CI					
0	4490	120	2.67	Ref	Ref
1	682	27	3.96	1.50 (0.98, 2.30)	1.43 (0.93, 2.20)
2	880	43	4.89	1.87 (1.31, 2.67)	1.78 (1.24, 2.56)
3+	630	26	4.13	1.57 (1.02, 2.42)	1.46 (0.94, 2.26)
EFI					
0-4	4184	112	2.68	Ref	Ref
5-8	2064	75	3.63	1.37 (1.02, 1.85)	1.31 (0.96, 1.78)
9-12	393	26	6.62	2.58 (1.66, 4.00)	2.26 (1.42, 3.62)
13+	41	<5**			
Comorbid diseases					
0	1901	53	2.79	Ref	Ref
1	2286	53	2.32	0.83 (0.56, 1.22)	0.80 (0.54, 1.18)
2	1430	57	3.99	1.45 (0.99, 2.12)	1.41 (0.96, 2.08)
3+	1065	53	4.98	1.83 (1.24, 2.69)	1.74 (1.16, 2.61)
Prescriptions					
0-4	1963	49	3.09	Ref	Ref
5-7	1832	55	2.75	1.21 (0.82, 1.79)	1.17 (0.79, 1.73)
8-12	1992	68	3.53	1.38 (0.95, 2.00)	1.28 (0.88, 1.87)
13+	895	33	3.67	2.02 (1.33, 3.06)	1.85 (1.21, 2.83)
PC contacts					
0-7	2167	67	3.09	Ref	Ref
8-11	1598	44	2.75	0.89 (0.60, 1.31)	0.85 (0.58, 1.25)
12-17	1528	54	3.53	1.15 (0.80, 1.65)	1.09 (0.76, 1.58)
18+	1389	51	3.67	1.19 (0.82, 1.73)	1.07 (0.74, 1.57)
Total	6682	216			

*Adjusted for age, gender, region of UK and calendar year of surgery
** The rules set for approval of use of CPRD data require that authors are not allowed to indicate the number of events in any category if they are less than 5

For peer review only

Table 5: Association between pre-operative multimorbidity and 90-day mortality

	No. at risk	Events	% Events	Unadjusted OR	Adjusted OR*
Charlson CI					
0	4490	32	0.71	Ref	Ref
1	682	6	0.88	1.24 (0.52, 2.97)	1.09 (0.45, 2.65)
2	880	13	1.48	2.09 (1.09, 4.00)	1.85 (0.95, 3.60)
3+	630	6	0.95	1.34 (0.56, 3.22)	1.12 (0.46, 2.73)
EFI					
0-4	4184	27	0.65	Ref	Ref
5-8	2064	20	0.97	1.51 (0.84, 2.69)	1.33 (0.72, 2.44)
9-12	393	10	2.54	4.02 (1.93, 8.37)	2.78 (1.24, 6.23)
13+	41	0	0	-	-
Comorbid diseases					
0	1901	14	0.74	Ref	Ref
1	2286	10	0.44	0.59 (0.26, 1.34)	0.58 (0.26, 1.33)
2	1430	16	1.12	1.53 (0.74, 3.14)	1.50 (0.71, 3.16)
3+	1065	17	1.60	2.19 (1.07, 4.45)	2.12 (0.99, 4.51)
Prescriptions					
0-4	1963	11	0.56	Ref	Ref
5-7	1832	19	1.04	1.86 (0.88, 3.92)	1.80 (0.85, 3.82)
8-12	1992	18	0.90	1.62 (0.76, 3.43)	1.39 (0.64, 2.98)
13+	895	9	1.01	1.80 (0.74, 4.37)	1.51 (0.61, 3.75)
Primary Care contacts					
0-7	2167	20	0.92	Ref	Ref
8-11	1598	13	0.81	0.88 (0.44, 1.78)	0.81 (0.40, 1.65)
12-17	1528	15	0.98	1.06 (0.54, 2.09)	0.90 (0.45, 1.78)
18+	1389	9	0.65	0.70 (0.32, 1.54)	0.53 (0.24, 1.19)
Total	6682	57			

*Adjusted for age, gender, region of UK and calendar year of surgery

For peer review only

Table 6: Patient Reported Outcome Measures (PROMs)

	N	Mean (SD)
Oxford Hip Score		
Pre-operative	1460	17.9 (7.9)
Post-operative	1416	39.3 (8.2)
Change	1402	21.3 (9.7)
EuroQoL Index (EQ-5D)		
Pre-operative	1380	0.35 (0.31)
Post-operative	1367	0.80 (0.23)
Change	1285	0.45 (34)

For peer review only

Table 7: Post-operative Oxford Hip Score by multimorbidity

	No.	Pre-op	Post-op	Change	Unadjusted difference	Adjusted*
Charlson CI						
0	960	18.5 (8.0)	39.9 (8.0)	21.4 (9.6)	Ref	Ref
1	113	17.6 (8.4)	37.5 (9.7)	20.1 (10.5)	-2.0 (-3.6, -0.5)	-2.0 (-3.6, -0.5)
2	199	16.0 (7.5)	37.8 (8.3)	21.9 (9.4)	-1.4 (-2.6, -0.2)	-1.0 (-2.2, 0.2)
3+	130	16.8 (7.1)	38.5 (8.1)	21.5 (10.0)	-1.15 (-2.5, 0.4)	-0.60 (-2.0, 0.8)
EFI						
0-4	798	18.8 (7.9)	40.7 (7.5)	21.8 (9.4)	Ref	Ref
5-8	511	17.3 (7.9)	37.7 (8.8)	20.5 (10.2)	-2.5 (-3.4, -1.7)	-2.0 (-2.9, -1.2)
9-12	86	14.6 (7.0)	35.8 (8.1)	21.4 (8.8)	-3.6 (-5.4, -1.9)	-2.9 (-4.7, -1.1)
13+	7	14.7 (8.2)	39.1 (10.4)	24.4 (8.7)	-0.45 (-6.2, 5.3)	1.0 (-4.8, 6.7)
Comorbid dis						
0	358	18.9 (7.8)	40.7 (7.9)	21.7 (9.9)	Ref	Ref
1	481	18.6 (8.1)	39.9 (7.9)	21.3 (9.6)	-0.7 (-1.8, 0.4)	-0.4 (-1.5, 0.6)
2	324	17.6 (7.9)	38.7 (8.2)	21.0 (9.6)	-1.6 (-2.8 , -0.4)	-1.2 (-2.3, 0.1)
3+	239	15.7 (7.5)	36.9 (8.9)	21.2 (9.7)	-3.0 (-4.3, -1.7)	-2.3 (-3.6, -1.0)
Prescriptions						
0-4	407	19.9 (7.4)	41.4 (7.1)	21.4 (9.3)	Ref	Ref
5-7	383	19.4 (8.1)	40.4 (7.4)	20.9 (9.5)	-0.8 (-1.9, 0.3)	-0.5 (-1.6, 0.6)
8-12	406	16.6 (7.7)	38.2 (8.5)	21.7 (9.9)	-2.3 (-3.4, -1.2)	-2.0 (-3.0, -0.9)
13+	206	14.1 (7.3)	35.4 (9.2)	21.3 (10.3)	-4.6 (-5.9, -3.2)	-4.0 (-5.3, -2.4)
PC contacts						
0-7	2167	18.9 (7.8)	39.9 (7.8)	21.0 (8.9)	Ref	Ref
8-11	1598	18.3 (8.1)	40.1 (7.8)	21.7 (10.0)	0.3 (-0.8 , 1.5)	0.6 (-0.6, 1.7)
12-17	1528	17.8 (7.8)	39.4 (8.1)	21.7 (9.9)	-0.1 (-1.2, 1.0)	0.4 (-0.7, 1.5)
18+	1389	16.1 (9.1)	37.2 (9.2)	21.1 (10.3)	-2.0 (-3.2, -0.8)	-1.5 (-2.7, -0.3)
Total	1402	1460	1416	1402		

*Adjusted for age, gender, region of UK and calendar year of surgery

For peer review only - http://bmjopen.bmj.com/site/about/guidelines.xhtml

Table 8: Post-operative EQ5D by multimorbidity

	No.	Pre-op	Post-op	Change	Unadjusted difference	Adjusted difference*
Charlson CI						
0	880	.37 (.31)	.82 (.22)	.45 (.34)	Ref	Ref
1	108	.33 (.32)	.77 (.25)	.44 (.33)	-.036 (-.080, .007)	-.037 (-.080, .007)
2	178	.29 (.31)	.76 (.22)	.48 (.34)	-.038 (-.073, -.002)	-.028 (-.064, .007)
3+	119	.33 (.29)	.74 (.24)	.40 (.33)	-.072 (-.114, -.030)	-.060 (-.102, -.018)
EFI						
0-4	735	.39 (.31)	.83 (.20)	.45 (.33)	Ref	Ref
5-8	464	.32 (.31)	.76 (.23)	.45 (.36)	-.059 (-.084, -.033)	-.048 (-.073, -.022)
9-12	79	.20 (.29)	.69 (.28)	.47 (.34)	-.141 (-.192, -.090)	-.126 (-.178, -.074)
13+	7	.20 (.35)	.78 (.35)	.59 (.32)	-.021 (-.184, .141)	.004 (-.157, .166)
Comorbid dis						
0	330	.39 (.31)	.85 (.19)	.46 (.34)	Ref	Ref
1	429	.38 (.31)	.81 (.20)	.43 (.32)	-.037 (-.068, -.005)	-.030 (-.062, .001)
2	306	.33 (.31)	.78 (.23)	.45 (.35)	-.060 (-.094, -.026)	-.049 (-.084, -.015)
3+	220	.26 (.32)	.71 (.28)	.45 (.36)	-.122 (-.159, -.084)	-.105 (-.143, -.067)
Prescriptions						
0-4	378	.42 (.30)	.85 (.20)	.44 (.34)	Ref	Ref
5-7	351	.41 (.30)	.83 (.20)	.42 (.32)	-.023 (-.055, .008)	-.062 (-.062, .001)
8-12	372	.30 (.30)	.77 (.22)	.47 (.34)	-.071 (-.103, -.040)	-.084 (-.084, -.015)
13+	184	.19 (.31)	.69 (.28)	.50 (.37)	-.139 (-.178, -.099)	-.143 (-.143, -.067)
PC contacts						
0-7	2167	.39 (.31)	.82 (.22)	.44 (.33)	Ref	Ref
8-11	1598	.36 (.30)	.83 (.21)	.46 (.34)	.003 (-.029, .036)	.007 (-.025, .039)
12-17	1528	.36 (.31)	.80 (.21)	.44 (.35)	-.018 (-.049, .013)	-.010 (-.042, .021)
18+	1389	.26 (.32)	.72 (.26)	.47 (.35)	-.079 (-.114, -.044)	-.070 (-.106, -.034)
Total	1285	1380	1367	1285		

*Adjusted for age, gender, region of UK and calendar year of surgery

For peer review only - http://bmjopen.bmj.com/site/about/guidelines.xhtml

Figure: Flow chart of available data

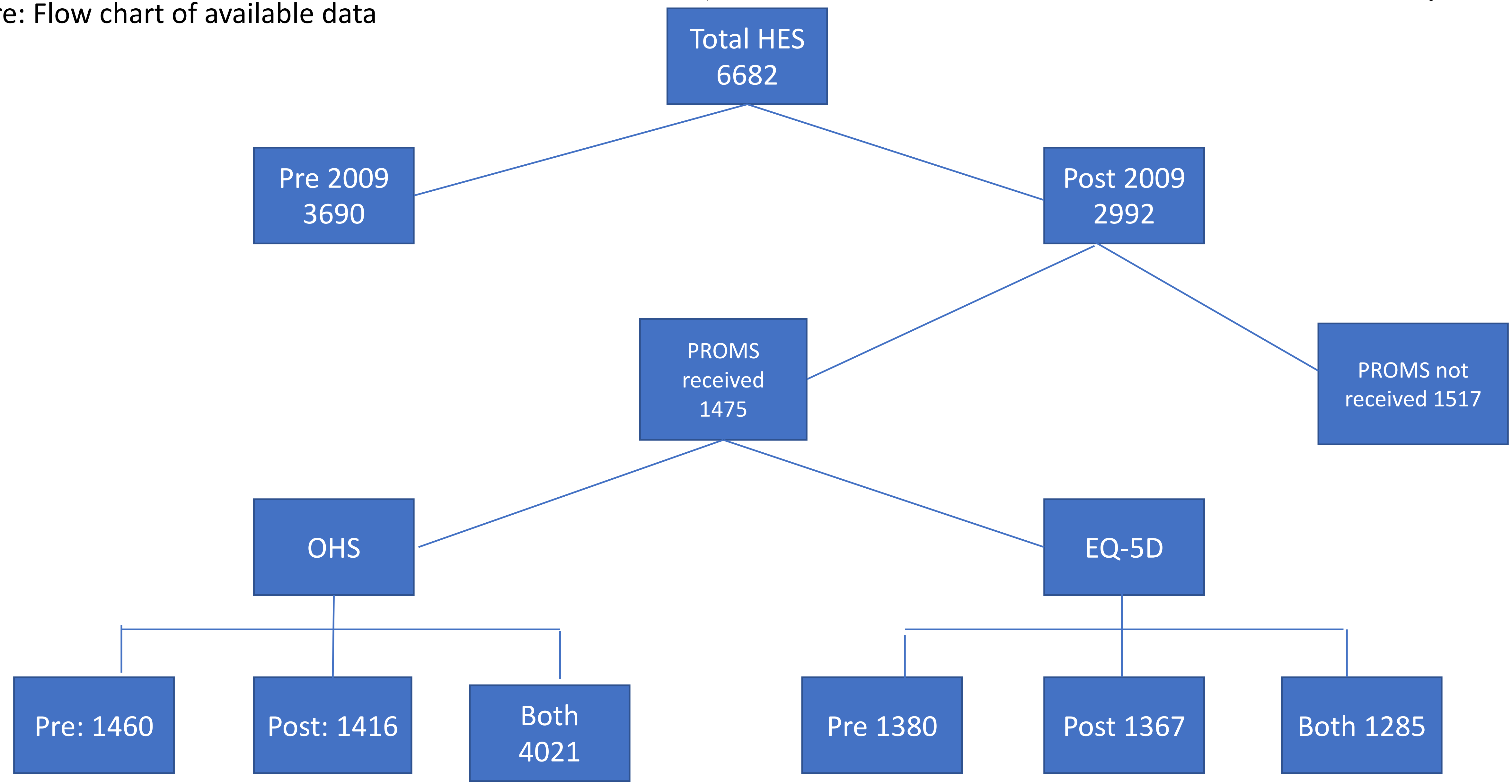

Supplementary Table: Baseline data on measures of pre-operative health status (multimorbidity, frailty and morbidity burden) in patients who underwent primary THR in HES APC during the date period in which PROMs were collected (N=2992)

	PROMs data	No PROMs data
**Charlson comorbidity index**		
0	1,013 (68.7)	931 (61.4)
1	123 (8.3)	152 (10.0)
2	205 (13.9)	254 (16.7)
3+	134 (9.1)	180 (11.9)
**Electronic frailty index (eFI)**		
0-4	831 (56.3)	752 (49.6)
5-8	544 (36.9)	593 (39.1)
9-12	93 (6.3)	151 (10.0)
13+	7 (0.5)	21 (1.4)
**No. comorbid diseases**		
0	378 (25.6)	320 (21.1)
1	497 (33.7)	464 (30.6)
2	346 (23.5)	364 (24.0)
3+	254 (17.2)	369 (24.3)
**No. medications prescribed**		
0-4	428 (29.0)	400 (26.4)
5-7	401 (27.2)	375 (24.7)
8-12	429 (29.1)	500 (33.0)
13+	217 (14.7)	242 (16.0)
**No. contacts with primary care**		
0-7	471 (31.9)	464 (30.6)
8-11	335 (22.7)	358 (23.6)
12-17	384 (26.0)	334 (22.0)
18+	285 (19.3)	361 (23.8)
**Total**	1475	1517

	PROMs data	*No PROMs data*
IMD		
1 (Affluent)	*414 (28.1)*	*389 (25.6)*
2	*374 (25.4)*	*369 (24.3)*
3	*356 (24.1)*	*367 (24.2)*
4	*239 (16.2)*	*255 (16.8)*
5 (Deprived)	*92 (6.2)*	*137 (9.0)*

For peer review only

		Reporting Item	Page Number
**Title and abstract**			
Title	#1a	Indicate the study's design with a commonly used term in the title or the abstract	1
Abstract	#1b	Provide in the abstract an informative and balanced summary of what was done and what was found	2
**Introduction**			
Background / rationale	#2	Explain the scientific background and rationale for the investigation being reported	4
Objectives	#3	State specific objectives, including any prespecified hypotheses	4
**Methods**			
Study design	#4	Present key elements of study design early in the paper	4
Setting	#5	Describe the setting, locations, and relevant dates, including periods of recruitment, exposure, follow-up, and data collection	4-5
Eligibility criteria	#6a	Give the eligibility criteria, and the sources and methods of selection of participants. Describe methods of follow-up.	5
Eligibility criteria	#6b	For matched studies, give matching criteria and number of exposed and unexposed	NA
Variables	#7	Clearly define all outcomes, exposures, predictors, potential confounders, and effect modifiers. Give diagnostic criteria, if applicable	5
Data sources / measurement	#8	For each variable of interest give sources of data and details of methods of assessment (measurement). Describe comparability of assessment methods if there is more than one group. Give information separately for exposed and unexposed groups if applicable.	5
Bias	#9	Describe any efforts to address potential sources of bias	8-10
Study size	#10	Explain how the study size was arrived at	6
Quantitative variables	#11	Explain how quantitative variables were handled in the analyses. If applicable, describe which groupings were chosen, and why	5,6
Statistical methods	#12a	Describe all statistical methods, including those used to control for confounding	5,6
Statistical methods	#12b	Describe any methods used to examine subgroups and interactions	NA
Statistical methods	#12c	Explain how missing data were addressed	7
Statistical methods	#12d	If applicable, explain how loss to follow-up was addressed	NA
Statistical methods	#12e	Describe any sensitivity analyses	7,8
**Results**			

For peer review only

Participants	#13a	Report numbers of individuals at each stage of study—eg numbers potentially eligible, examined for eligibility, confirmed eligible, included in the study, completing follow-up, and analysed. Give information separately for exposed and unexposed groups if applicable.	Figure
Participants	#13b	Give reasons for non-participation at each stage	Figure
Participants	#13c	Consider use of a flow diagram	Figure
Descriptive data	#14a	Give characteristics of study participants (eg demographic, clinical, social) and information on exposures and potential confounders. Give information separately for exposed and unexposed groups if applicable.	17
Descriptive data	#14b	Indicate number of participants with missing data for each variable of interest	17
Descriptive data	#14c	Summarise follow-up time (eg, average and total amount)	6
Outcome data	#15	Report numbers of outcome events or summary measures over time. Give information separately for exposed and unexposed groups if applicable.	7-9
Main results	#16a	Give unadjusted estimates and, if applicable, confounder-adjusted estimates and their precision (eg, 95% confidence interval). Make clear which confounders were adjusted for and why they were included	20-24
Main results	#16b	Report category boundaries when continuous variables were categorized	20-24
Main results	#16c	If relevant, consider translating estimates of relative risk into absolute risk for a meaningful time period	NA
Other analyses	#17	Report other analyses done—e.g., analyses of subgroups and interactions, and sensitivity analyses	25
**Discussion**			
Key results	#18	Summarise key results with reference to study objectives	8
Limitations	#19	Discuss limitations of the study, taking into account sources of potential bias or imprecision. Discuss both direction and magnitude of any potential bias.	8-10
Interpretation	#20	Give a cautious overall interpretation considering objectives, limitations, multiplicity of analyses, results from similar studies, and other relevant evidence.	10,11
Generalisability	#21	Discuss the generalisability (external validity) of the study results	11
**Other Information**			
Funding	#22	Give the source of funding and the role of the funders for the present study and, if applicable, for the original study on which the present article is based	12

Statement: STROBE guidelines for reporting observational studies.

For peer review only

For peer review only

For peer review only - http://bmjopen.bmj.com/site/about/guidelines.xhtml

BMJ Open

Does pre-existing morbidity influence risks and benefits of total hip replacement for osteoarthritis: a prospective study of 6682 patients from linked national datasets in England?

Journal:	*BMJ Open*
Manuscript ID	bmjopen-2020-046712.R1
Article Type:	Original research
Date Submitted by the Author:	22-Mar-2021
Complete List of Authors:	Ferguson, Rory; University of Oxford Nuffield Department of Orthopaedics Rheumatology and Musculoskeletal Sciences, Botnar Institute<br>Prieto-Alhambra, Daniel; University of Oxford, Centre for Statistics in Medicine, NDORMS<br>Peat, George; Keele University, Research Institute for Primary Care & Health Sciences<br>Delmestri, Antonella; University of Oxford, Nuffield Department of Orthopaedics, Rheumatology, and Musculoskeletal Sciences<br>Jordan, Kelvin; Keele University, School of Primary, Community and Social Care<br>Strauss, Vicky; University of Oxford, Centre for Statistics in Medicine, Ndorms<br>Valderas, Jose; University of Exeter Medical School,<br>Walker, Christine; Keele University, Research User Group, Arthritis Research UK Primary Care Centre, Research Institute of Primary Care and Health Sciences<br>Yu, Dahai; Keele University, Research Institute for Primary Care & Health Sciences<br>Glyn-Jones, Sion; University of Oxford, NDORMS<br>Silman, Alan; University of Oxford Nuffield Department of Orthopaedics Rheumatology and Musculoskeletal Sciences, Botnar Institute
**Primary Subject Heading**:	Surgery
Secondary Subject Heading:	Geriatric medicine, Rheumatology
Keywords:	GERIATRIC MEDICINE, SURGERY, Hip < ORTHOPAEDIC & TRAUMA SURGERY, ORTHOPAEDIC & TRAUMA SURGERY

SCHOLARONE™
Manuscripts

I, the Submitting Author has the right to grant and does grant on behalf of all authors of the Work (as defined in the below author licence), an exclusive licence and/or a non-exclusive licence for contributions from authors who are: i) UK Crown employees; ii) where BMJ has agreed a CC-BY licence shall apply, and/or iii) in accordance with the terms applicable for US Federal Government officers or employees acting as part of their official duties; on a worldwide, perpetual, irrevocable, royalty-free basis to BMJ Publishing Group Ltd ("BMJ") its licensees and where the relevant Journal is co-owned by BMJ to the co-owners of the Journal, to publish the Work in this journal and any other BMJ products and to exploit all rights, as set out in our licence.

The Submitting Author accepts and understands that any supply made under these terms is made by BMJ to the Submitting Author unless you are acting as an employee on behalf of your employer or a postgraduate student of an affiliated institution which is paying any applicable article publishing charge ("APC") for Open Access articles. Where the Submitting Author wishes to make the Work available on an Open Access basis (and intends to pay the relevant APC), the terms of reuse of such Open Access shall be governed by a Creative Commons licence – details of these licences and which Creative Commons licence will apply to this Work are set out in our licence referred to above.

Other than as permitted in any relevant BMJ Author's Self Archiving Policies, I confirm this Work has not been accepted for publication elsewhere, is not being considered for publication elsewhere and does not duplicate material already published. I confirm all authors consent to publication of this Work and authorise the granting of this licence.

For peer review only

Does pre-existing morbidity influence risks and benefits of total hip replacement for osteoarthritis: a prospective study of 6682 patients from linked national datasets in England

RJ Ferguson [1]
D Prieto-Alhambra [1]
G Peat [2]
A Delmestri [1]
KP Jordan [2]
V Strauss [1]
JM Valderas [3]
C Walker [2]
D Yu[2]
S Glyn-Jones [1]
AJ Silman[1]

Authorship Statement: The study was conceived by AJS with SG-J and DP-A and detailed protocol produced by these authors with input from GP, KPJ, JMV and DY. AD was responsible for extracting, preparing and ensuring the quality of the datasets for analysis; analysis was undertaken by RJF supported by VS, DP-A and AJS, CW provided patient input into the design, approach to analysis and interpretation of the data. All authors contributed to the discussion of the results. The manuscript was prepared by AJS and RJF and reviewed by all authors

[1]Nuffield Department of Orthopaedics, Rheumatology and Musculoskeletal Sciences, University of Oxford, Oxford, UK.
[2]Primary Care and Health Sciences Department, Keele University, Keele, UK.
[3]University of Exeter Medical School, Exeter, UK.
[4]Oxford University Hospital NHS Foundation Trust, Oxford, UK.

Correspondence to Professor Silman
Nuffield Department of Orthopaedics, Rheumatology and Musculoskeletal Sciences,
University of Oxford
Botnar Research Centre, Windmill Road
Headington, Oxford, OX3 7LD.
alan.silman@ndorms.ox.ac.uk

Word count abstract 300, main paper 3517

Key words: Hip osteoarthritis, arthroplasty, multimorbidity, mortality, outcome, quality of life

For peer review only - http://bmjopen.bmj.com/site/about/guidelines.xhtml

For peer review only

Funding

This report is independent research funded by the National Institute for Health Research (Research for Patient Benefit programme, PB-PG-0815-20024). The views expressed in this publication are those of the authors and not necessarily those of the NIHR or the Department of Health and Social Care

This study is based in part on data from the Clinical Practice Research Datalink (CPRD) obtained under licence from the UK Medicines and Healthcare products Regulatory Agency. The data is provided by patients and collected by the NHS as part of their care and support. The interpretation and conclusions contained in this study are those of the author/s alone
HES Data/ONS Data shown are Copyright © 2016 are re-used with the permission of The Health & Social Care Information Centre. All rights reserved.
The OPCS Classification of Interventions and Procedures, codes, terms and text is Crown copyright (2016) published by Health and Social Care Information Centre, also known as NHS Digital and licensed under the Open Government Licence available at www.nationalarchives.gov.uk/doc/open-government-licence/open-government-licence.htm.

Ethical Approval

No ethical approval was required and anonymised data were provided by as above. No individual patient consent was therefore required

Consent for publication: not applicable

Availability of data and materials: The data that support the findings of this study are available from CPRD but restrictions apply to the availability of these data, which were used under license for the current study, and so are not publicly available. The datasets extracted from CPRD and used in the analysis during the current study are available from the corresponding author on reasonable request and consistent with the permission received from CPRD

Conflicts of interests:

The authors declare that they have no conflicts of interests to report

Acknowledgements

We acknowledge Professor Andy Judge for advice on statistical analysis at the earliest stages of this project . We are also grateful to Professor Andrew Clegg, and Stephen Pye for guidance in the use of the electronic frailty index. Dr John Griffiths and Professor Nigel Arden, provided very helpful advice at the design stage on the challenges of anaesthetic assessment and on studying osteoarthritis from an epidemiological perspective..

For peer review only - http://bmjopen.bmj.com/site/about/guidelines.xhtml

Abstract

Background

Total hip arthroplasty (THA) for elderly people with multimorbidity increases the risk of serious health hazards including mortality. Whether background morbidity reduces the clinical benefit is less clear

Objective

To evaluate how pre-existing health status, using multiple approaches, influences the risks of, and quality of life benefits from, THA.

Design

Longitudinal record linkage study

Setting

UK national sample of linked primary and secondary care registers.

Participants

All 6682 patients, between 1995 and 2014, with hip osteoarthritis identified from the national primary care register who had a THA. Data from their primary care record was extracted to score 5 different constructs: Charlson Comorbidity Index, Electronic Frailty Index (eFI) and counts of comorbidity disorders (from list of 17), prescribed medications and number of primary care visits

Outcome measures

Linked secondary care registers provided data on post-operative mortality and major medical complications. Linkage to the national Patients Reported Outcome Measures (PROMs) database permitted assessment of change in hip-specific and general improvement in quality of life using the Oxford Hip Score (OHS) and EQ-5D respectively

Results

Peri-operative complication rate was 3.2% and mortality was 0.9%, both increased with worse pre-operative health status but with only modest increases in the highest risk strata: all adjusted odd ratios (OR) under 2.0 exception being eFI: most frail OR=4.3; 95%CI [0.95-19.7]). By contrast, the benefits were not reduced in those with more adverse health: PROMs improvement with either OHS or EQ-5D, was similar across morbidity strata. There were differences in the results depending on the approach to measuring pre-operative health

Conclusions

Independent of pre-operative morbidity, THA leads to similar substantial improvements in quality of life. These are offset by an increase in medical complications in the subgroup of patients with high morbidity. For most elderly people, their other health disorders should not be a barrier for THA.

(300 words)

For peer review only - http://bmjopen.bmj.com/site/about/guidelines.xhtml

Strengths and limitation

- National large sample of older patients recorded in primary care with newly reported osteoarthritis of the hip and who had received a hip replacement
- Multi-dimensional approach to assessing their concurrent morbidity and health status prior to surgery from the primary care record
- Linkage to their surgical outcomes both hazards - mortality and significant post-operative complications and the benefits - improvement in quality of life
- Such study using routine national datasets have advantage of size, access to complete health record and linkage to both data hazards and benefits from surgery
- Relying on routine data has the challenges of completeness of recording and accuracy of the data items extracted

For peer review only

Background

National data on the short term outcome from total hip arthroplasty (THA) demonstrate a substantial improvement in quality of life.[1] Most recipients are older adults: the median age for example in the UK countries reporting to the National Joint Registry is 69.[2]

With increasing age, patients are increasingly likely to develop multiple chronic health disorders (multimorbidity) with many patients described as frail.[3–7] Such disorders increase the hazards from surgery[8–10] and may also limit the benefits in quality of life.[11–13] There is in practice an inevitable selection process prior to surgical acceptance of older adults with multimorbidity, though there is no specific guidance on how these factors should influence surgical decision making, either in UK[14] or USA.[15]

There are challenges in identifying a valid approach to measure the cumulative severity of all chronic health disorders.[16] Simple counts of the presence of specific chronic diseases, or weighted instruments, typically focus on disorders associated with the hazards but exclude those that might impact on quality of life. In a companion paper to this one we have demonstrated that in current practice in the UK, pre-existing health problems, even when only moderate in effect, do influence the likelihood and timing of THA. The question that then arose is whether this impact on acceptance for surgery appropriately reflects how far these health issues impact on surgical outcome.[17]

Our goal was to identify the impact that multimorbidity and frailty on the risks and benefits of THA. Our specific objectives were to assess, using a number of different approaches to scoring, how multimorbidity influenced the risks and benefits of elective total hip replacement for osteoarthritis. The former outcome was assessed by post-operative complications, length of stay and hospital readmission; and the latter assessed by patient reported outcome scores (PROMs) covering pain, function and quality of life. To maximise the external validity, the study took advantage of the availability of English national datasets linking primary and secondary care.

Methods

Summary of design

In a longitudinal record linkage study using a national database, patients with a newly recorded diagnosis of hip osteoarthritis were identified from their primary care record. Linkage to the national secondary care database identified those individuals who had a THA, who became the subjects of the study. The primary care record was interrogated to provide information on other health disorders and treatments were used to derive measures of pre-existing multimorbidity and health status at the time of surgery. This secondary care database also provided data on peri-operative death and major complications as well as scores of patient outcome. The influence of the scores from these measures on mortality, in-

For peer review only - http://bmjopen.bmj.com/site/about/guidelines.xhtml

patient complications and readmission within 90 days and PROMs at 6 months were then calculated.

Data sources

We used the UK Clinical Practice Research Datalink (CPRD) which contains the primary care electronic medical records of approximately 4.4 million active patients and representative of the wider UK population.[18] Data are stored with Read codes for diseases that are cross-referenced to the International Classification of Diseases (ICD-10).

The Hospital Episode Statistics dataset (HES) contains all patient attendances at NHS hospitals in England and covers episodes of care. HES data which were provided anonymously records patient demographic data, diagnoses and procedures and is linked to national death certificate data. HES has also been linked since 2009 to the PROMs database gathering outcome data NHS-funded THA both pre-operatively and at 6 months post-operatively.[1]

Study Population

All patients in the CPRD with a READ code for hip osteoarthritis from January 1 1995, until March 31, 2014, and aged 65 years or over at the time of diagnosis, were identified (ISAC protocol number 17_024R). The validation of those Read codes for hip osteoarthritis are described elsewhere.[19] Those patients who underwent elective primary THA, identified from HES were included in the analysis. Operations were identified from the Classification of Surgical Operations and Procedures (OPCS-4) codes.

The following measures of multimorbidity prior to the date of surgery were extracted from the accumulated primary care record. These were: (i) Charlson Comorbidity Index (CCI)-developed to predict 1-year mortality based on the presence of specified chronic diseases.[20], (ii) count of the 17 chronic diseases listed in the NHS Quality Outcomes Framework (QOF).[21] (iii) electronic Frailty Index (eFI), a score based on the cumulative deficit model of frailty and validated against mortality, hospitalisation and nursing home admission.[22] We also used 2 measures of the burden of care associated with the presence of chronic conditions. Thus, within the 12 months prior to surgery we extracted the numbers of (i) different prescribed medications and (ii) primary care contacts for any reason.

Outcomes

All subjects had the following outcomes, in addition to death, extracted from the linked HES data within 90 days of THA: venous thromboembolism, myocardial infarction, stroke, anaemia, lower respiratory tract infection, urinary tract infection, wound infection. Also extracted were length of stay following surgery and readmission after discharge within 90 days. The national PROMs database provided data since 2009 on (i) Oxford Hip Score as a measure of hip function and (ii) EQ-5D Index as a measure of quality of life at 6 months post-operatively on all patients receiving a THA – information on the appropriate interpretation of

For peer review only - http://bmjopen.bmj.com/site/about/guidelines.xhtml

these scores is provided in the Appendix. Although all patients are invited to complete the PROMs questionnaires pre-operatively and at 6 months post-operatively. Completion rates vary, plus there are some delays in those who do respond beyond the 6 months.[1]

Statistical analysis

The scores for each of the five measures of multimorbidity were split into 4 categories. The eFI has 4 categories defined by the developers: fit (0-4), mild frailty (5-8), moderate frailty (9-12), and severe frailty (13+). The actual CCI scores were used to split into categories of score of 0, 1, 2, and 3+. Based on the distribution of the data the counts of number of chronic diseases were also split in categories of score of 0, 1, 2, and 3+. The categories for the number of medications prescribed and of primary care visits were derived from the observed distributions of the actual data, aiming for equal sized quarters: with medication counts divided into scores of 0-4, 5-7, 8-12, and 13+ and number of primary care visits into 0-7, 8-11, 12-17 and 18+. The lowest category for each score was used as the referent category in the analysis.

Logistic regression methods were used to calculate the unadjusted odds of a complication or readmission to hospital by multimorbidity category. Poisson regression methods were used to calculate the unadjusted difference in length of stay by multimorbidity category. Linear regression methods were used to calculate the difference in post-operative OHS and EQ-5D score, adjusted for pre-operative score, by multimorbidity category. All models were then adjusted for age at surgery, sex, region of the UK, and calendar year of total hip replacement.

Patient and Public involvement

The research question was informed by our patient representatives both in Oxford and Keele, who helped shape the objectives and the outcome measures. One of the representatives, (author CW) contributed to the planning of the analysis. The Research Users group at Keele engaged in discussions about the results, their interpretation and dissemination. As this was a database study, there was not patient involvement in recruitment or data collection

Results

Baseline data

The demographic characteristics of the study participants are shown in Table 1. There were 6682 patients with a code for hip osteoarthritis on the primary care record who after linkage to the HES data, had had a THA within the follow up period from April 1997 to March 2014. The mean age at surgery was 76 years (SD 6).

Pre-operative scores for the four multimorbidity measures derived are shown in Table 2. Only 20% had a CCI above 1 perhaps reflecting the selection of patients for surgery. Based on the

For peer review only - http://bmjopen.bmj.com/site/about/guidelines.xhtml

eFI classification, 34% were classified as 'mildly frail' and 6.5% 'moderately frail'. Around 1 in 6 had 3 or more chronic disorders recorded. Over 40% had been prescribed at least 8 medications, and a similar proportion had more than 12 primary care visits, in the previous 12 months.

Outcomes

Data on complications, length of stay and readmission to hospital were available on all patients (as linkage to HES was a criterion for study entry). As mentioned above, data on PROMs was only available on a subset of patients who completed the questionnaires (Figure). In total, pre- and post-operative OHS was available on 1402 patients, and pre- and post-operative EQ-5D Index was available on 1285 patients. As a test of bias, we compared the multimorbidity measures between those with and without PROMs (Supplementary Table 1) which whilst showing no substantive shifts, across all the measures, those who did not provided PROMS were more likely to have worse multimorbidity and also reside in more socio economically deprived areas.

Association between multimorbidity measures and adverse outcomes

In all, 216 (3.2%) patients suffered a post-operative complication including thrombo-embolism, myocardial infarction, urine and respiratory infections and wound infection, and 57 (0.9%) patients died, within 90 days of surgery (Table 3). The rate of all-cause readmission to hospital within 90 days of surgery was 11%.

The impact of multimorbidity scores across all these medical complications are shown in Table 4. The overall adverse event rate was modestly increased with increasing levels of multimorbidity relative to the lowest scoring group. These results were similar result across the different approaches to assessing multimorbidity. Given the large numbers in the study the confidence intervals were relatively narrow. The actual increases in risks were similarly modest, both before and after age adjustment. Those in highest frailty group were the most predictive, compared to the other measures of multimorbidity, of a medical complication. By contrast the number of prescribed medications had little influence on that outcome. We analysed the individual complications separately (data not shown). The numbers with any of the individual complications were modest but there were no substantial difference between the risks of individual complications in their relationship with the multimorbidity scores.

The data on mortality are shown in Table 5. The mortality risk in the least healthy stratum ranged from 0.7% for primary care contacts to 2.3% for those with an eFI of 9 or more. There was an inconsistent association between death and Charlson score but the number of events in those greater than score of 2 were too small for useful analysis. Interestingly the category of count of medications prescribed did not affect the mortality rate. The measure with the greatest predictive power of death was the eFI. After adjustment for age and gender, those

For peer review only - http://bmjopen.bmj.com/site/about/guidelines.xhtml

in the two highest frailty groups had a 2.5-fold increased mortality risk. The overall number of deaths was modest and the resulting small numbers, when stratified by pre-operative morbidity category, limits the precision of these estimates.

The impact of multimorbidity scores on length of stay were also analysed. Detailed data are not shown but in brief for all the multimorbidity measures, apart from the eFI, there was no impact on length of stay. The median length of stays in the lowest and highest categories of multimorbidity were: 6 and 7 days for Charlson score, 6 and 8 days for eFI score, 6 and 7 days for the count of chronic diseases, and 6 and 7 days for the count of medications prescribed.

We have also examined the impact of these pre-operative health measures on 2 indirect measures of complications: readmission rates and length of stay (LOS). Overall, there were 786 patients readmitted for any reason over the next 90 days. Risks were related to level of pre-operative health with those in the worst health categories having a twofold increased risk compared to those in in the healthiest group. The results were broadly similar independent of the scoring approach (Supplementary Table 1). As regards LOS, the differences between grades of prior ill health were modest, with the median and interquartile ranges almost identical. The eFI was a slight outlier where those in the highest two categories had a median LOS two days higher than those with lesser frailty (Supplementary Table 2)

Association between multimorbidity measures and quality of life

Across all patients, there was an overall marked improvement in both the specific (OHS) and the generic (EQ-5D) patient outcomes. The summary outcome data are shown in Table 6, confirming substantial improvement at 6 months in both the OHS and EQ-5D across the population studied. The mean improvement in OHS of around 20 did not vary importantly between the different levels of any of the approaches to assessing the pre-operative morbidity (Table 7). For each of our measures of multimorbidity, the pre-operative OHS showed was lower with increasing score. What was of note was the similarity in the relative improvement in the post-operative OHS seen across the board for all measures of multimorbidity. Typically mean pre-operative scores in the mid to late teens reached scores in the high 30's and 40's: the highest score achievable is 48. Thus, surgery led to a marked improvement, not attenuated by the severity of the pre-operative general health.

A similar analysis was undertaken on EQ-5D (Table 8). As expected, those with a higher pre-operative morbidity had a lower pre-operative EQ-5D, reflecting the impact of the patients' other comorbid disorders on their overall quality of life. However, the 6-month incremental improvement in EQ-5D was almost identical across all categories of multimorbidity, independent of the method of multimorbidity scoring. The actual EQ-5D scores were moderately lower in the highest categories of multimorbidity but the relative improvement

For peer review only

was the same. Regression analyses, both unadjusted and adjusted for age and gender showed that the post-operative scores, adjusted for the pre-operative scores, were not importantly different (see Appendix 1) for any of the multimorbidity scores compared with the lowest score in each grading system.

Discussion

This is the first study to comprehensively assess the impact of pre-operative health measures, on the risks and benefits of THA. We have demonstrated that there is a link between worse preoperative health status with the risk of death and of clinically significant medical event rates, although the size and the steepness of any trend varied between the different approaches to assessing health. There is no single obvious measure which captures all the relevant dimensions. Of interest was that these approaches in general did not yield consistent differences in these results, although the number of primary care contacts was 'an outlier' in this respect and would not appear to be useful as a measure of health status.

By contrast the other important result was that THA is associated with a substantial improvement in quality of life, independent of level of pre-existing multimorbidity. Indeed, this was true for all the approaches used to assess health.

There are some limitations of these data. Firstly, we have not considered the impact of different types of surgery and anaesthesia together with other prophylactic interventions to reduce the hazards from surgery in this group of patients. For example, the routine use of the use of thromboprophylaxis should reduce the risk of thromboembolism even in those with the highest multimorbidity. Such interventions would not, though, explain the observation that improvement in patient outcome is not materially affected by pre-exiting morbidity.

Secondly, inevitably in studies of this type, there is a reliance on the completeness, and quality diagnostic accuracy in the data sets available. We relied on the quality of the CPRD to provide information on the diagnosis of osteoarthritis of the hip and of the multimorbidity scores. As regards the former, a validation study using radiological and clinical information from a subsample of this cohort, described elsewhere, confirmed the diagnosis of hip osteoarthritis in 80 per cent.[19] As regards the latter there are many reports on the validity of the national datasets used for deriving multimorbidity information.[18,23]

There is also a similar reliance on the quality of the outcome data from HES. Although data from HES is widely accepted as being sufficiently valid,[24] we were concerned that this data source captured a high proportion of these relevant outcome events with minimal misclassification. As a test of the quality of the outcomes data, we therefore compared our results on post-operative hazard rates with other published data to determine if such rates

For peer review only - http://bmjopen.bmj.com/site/about/guidelines.xhtml

were broadly similar. There are limitations in such comparisons, but this analysis suggested that the observed rates of clinically significant events in the population studied did accord with those published from other countries. For example, our VTE incidence was 2.1%, published population data suggests a range of 0.7-3.9%.[25–27] Similar comparisons for MI were: present study 0.4%, literature 0.4-0.6%[28–30]; wound infection present study 1.7%, literature 2.2-2.9%.[31]

Readmission rate is also useful as an indirect measure of complications. Indeed, the impact of both being in the most frail category of eFI and number of prescriptions was similar on the rates of both readmissions (Supplementary Table 1) to that seen on complications (Table 4). The agreement was less observed with primary care contacts which reflects health seeking behaviours as well as morbidity.

Pre-operative health status had little effect on LOS, perhaps surprisingly but this may be complex to unpack given differences in anaesthetic procedure. We specifically studied multiple ways of measuring pre-existing morbidity and its impact on health, given there is no single 'best buy' and all such scores are measures of an underlying, but hard-to-measure, construct. This resulted in multiple estimates of effect. We were not attempting to prove statistical significance by finding one or more measure that was useful. More, by using multiple options, we covered as far as possible the different suggested approaches to ascertaining multimorbidity. These measures, whilst overlapping in the patients classified, were far from being concordant, but this is not surprising as they were measuring different constructs. Despite such differences, none of the scoring systems had substantial effects on outcome.

There are other variables that might be confounders which have not been addressed in the analyses presented which include socio-economic status, body mass index (BMI), and cigarette smoking. We specifically did not adjust for the possible confounding effects of these variables in part because of the quality and completeness of the data. More importantly, we were concerned that the effect of these variables would most likely be mediated by their association with other comorbid disorders and interpretation of such adjusted results would be difficult.

The data on benefits were only collected at 6 months post-operatively. We cannot comment on the longer- term impact of these pre-operative measures on quality of life. This is not without challenges given the increasing likelihood over time that there may be other changes in health that will impact on overall benefit.

Finally, the patients studied in this cohort were inevitably those who underwent surgery. Our comparisons therefore were between those in the different morbidity categories for whom a

For peer review only - http://bmjopen.bmj.com/site/about/guidelines.xhtml

decision had been made that surgery was appropriate. We could not address whether, *within each such category*, there were selective differences in surgical decision making that might be associated with the outcomes measured. One reasonable unmeasured confounder relates to prior perception of benefit. Thus, the individuals with major health issues who agree to surgery might have a higher expectation of outcome. Such individuals might then score higher on their self-reported outcomes, than otherwise similar patients who did not have surgery

This study aimed to investigate the influence of common long-term disorders in the population and their influence on the risks and benefits from hip surgery. It is accepted that those at greatest risk from anaesthesia and major surgery should be identified, especially for an elective procedure such as THA. Pre-operative morbidity does increase the mortality risk.[8,10,32] By contrast, there are very little data on how the co-existing health problems affect the clinical benefits. Indeed, we have shown in a recent analysis that those THA patients with American Society of Anesthesiologists (ASA) Physical Health Classification System[33] grade III or higher are at increased risk of revision surgery[34], a result that is not unexpected. ASA grades whilst routinely collected for example in national joint registers, are not extractable from primary care data systems. The underlying concern behind undertaking the current study was that, especially in older individuals, the lack of an evidence base weighing up the risks and benefits from surgery, leads to patients, their families and health care professionals, accepting that surgery should be avoided. The current study suggests that the increased risks are modest and the benefits similar.

As part of the study design, we have been able to examine a number of different approaches to assessing multimorbidity in primary care patients. Indeed, that setting with the complete medical record provides the only practical one for scoring patients in clinical practice. The eFI, a recently developed tool for assessing frailty from the primary care record, is achieving widespread usage in the UK and elsewhere for classifying frailty in elderly patients. High eFI scores are associated with substantial increased risks of mortality, unplanned hospital admission and the need for long term care. It is thus interesting that within the current dataset the differences in eFI class were, if anything more predictive of outcomes than the other approaches. It should be noted though that the proportion of the population selected for surgery in the highest eFI category was small. Further, even a high eFI class on our data, should not, of itself, be a barrier to surgery.

We conclude that in older patients requiring a hip replacement, unless they are in the small group whose anaesthetic risk precludes safe surgery, there is no evidence that their other long-term disorders should be a barrier to this life changing elective procedure.

For peer review only

References

1 NHS Digital. Provisional Quarterly Patient Reported Outcome Measures (PROMs) in England. 2017. https://digital.nhs.uk/catalogue/PUB23060.

2 National Joint Registry. National Joint Registry for England, Wales, Northern Ireland and the Isle of Man: 17th Annual Report. 2020, Table 3.H4: https://reports.njrcentre.org.uk/Portals/0/PDFdownloads/NJR%2017th%20Annual%20Report%202020.pdf.

3 Barnett K, Mercer SW, Norbury M, Watt G, Wyke S, Guthrie B. Epidemiology of multimorbidity and implications for health care, research, and medical education: a cross-sectional study. *Lancet* 2012; **380**: 37–43.

4 Violan C, Foguet-Boreu Q, Flores-Mateo G, *et al.* Prevalence, Determinants and Patterns of Multimorbidity in Primary Care: A Systematic Review of Observational Studies. *PLoS One* 2014; **9**: e102149.

5 Abad-Díez JM, Calderón-Larrañaga A, Poncel-Falcó A, *et al.* Age and gender differences in the prevalence and patterns of multimorbidity in the older population. *BMC Geriatr* 2014; **14**: 75.

6 Gale CR, Cooper C, Aihie Sayer A. Prevalence of frailty and disability: findings from the English Longitudinal Study of Ageing. *Age Ageing* 2015; **44**: 162–5.

7 Vetrano DL, Palmer K, Marengoni A, *et al.* Frailty and Multimorbidity: A Systematic Review and Meta-analysis. *Journals Gerontol Ser A* 2019; **74**: 659–66.

8 Aynardi M, Jacovides CL, Huang R, Mortazavi SMJ, Parvizi J. Risk Factors for Early Mortality Following Modern Total Hip Arthroplasty. *J Arthroplasty* 2013; **28**: 517–20.

9 Rasouli MR, Restrepo C, Maltenfort MG, Purtill JJ, Parvizi J. Risk Factors for Surgical Site Infection Following Total Joint Arthroplasty. *J Bone Jt Surgery-American Vol* 2014; **96**: e158-1–5.

10 Singh JA, Jensen MR, Harmsen WS, Gabriel SE, Lewallen DG. Cardiac and thromboembolic complications and mortality in patients undergoing total hip and total knee arthroplasty. *Ann Rheum Dis* 2011; **70**: 2082–8.

11 Gordon M, Paulsen A, Overgaard S, Garellick G, Pedersen AB, Rolfson O. Factors influencing health-related quality of life after total hip replacement - a comparison of data from the Swedish and Danish hip arthroplasty registers. *BMC Musculoskelet Disord* 2013; **14**: 316.

12 Greene ME, Rolfson O, Gordon M, Garellick G, Nemes S. Standard Comorbidity Measures Do Not Predict Patient-reported Outcomes 1 Year After Total Hip Arthroplasty. *Clin Orthop Relat Res* 2015; **473**: 3370–9.

13 Loth FL, Giesinger JM, Giesinger K, *et al.* Impact of Comorbidities on Outcome After Total Hip Arthroplasty. *J Arthroplasty* 2017; **32**: 2755–61.

14 National Institute for Health and Clinical Excellence. Osteoarthritis: Care and Management Guidelines. 2014. https://www.nice.org.uk/guidance/cg177.

15 American Academy of Orthopaedic Surgeons. Osteoarthritis of the Hip - Clinical Practice Guideline. https://www.aaos.org/quality/quality-programs/lower-extremity-programs/osteoarthritis-of-the-hip/ (accessed March 17, 2021).

16 The Academy of Medical Sciences. Multimorbidity: a priority for global health research. 2018 https://acmedsci.ac.uk/file-download/82222577 (accessed April 4,

For peer review only

2019).
17 Ferguson RJ, Prieto-Alhambra D, Peat G etal . Influence of pre-existing multimorbidity on receiving a hip arthroplasty: Cohort study of 28,026 elderly subjects from UK primary care. *(submitted)*
18 Herrett E, Gallagher AM, Bhaskaran K, *et al.* Data Resource Profile: Clinical Practice Research Datalink (CPRD). *Int J Epidemiol* 2015; **44**: 827–36.
19 Ferguson RJ, Prieto-Alhambra D, Walker C, *et al.* Validation of hip osteoarthritis diagnosis recording in the UK Clinical Practice Research Datalink. *Pharmacoepidemiol Drug Saf* 2018; **28**: 187–93.
20 Charlson ME, Pompei P, Ales KL, MacKenzie CR. A new method of classifying prognostic comorbidity in longitudinal studies: development and validation. *J Chronic Dis* 1987; **40**: 373–83.
21 NHS Digital. Quality and Outcomes Framework Programme. http://content.digital.nhs.uk/qof (accessed Oct 5, 2017).
22 Clegg A, Bates C, Young J, *et al.* Development and validation of an electronic frailty index using routine primary care electronic health record data. *Age Ageing* 2016; **45**: 353–60.
23 Jick SS, Kaye JA, Vasilakis-Scaramozza C, *et al.* Validity of the General Practice Research Database. Pharmacotherapy. 2003; **23**: 686–9.
24 Herbert A, Wijlaars L, Zylbersztejn A, Cromwell D, Hardelid P. Data Resource Profile: Hospital Episode Statistics Admitted Patient Care (HES APC). *Int J Epidemiol* 2017; **46**: 1093-1093i.
25 Senay A, Trottier M, Delisle J, *et al.* Incidence of symptomatic venous thromboembolism in 2372 knee and hip replacement patients after discharge: data from a thromboprophylaxis registry in Montreal, Canada. *Vasc Health Risk Manag* 2018; **Volume 14**: 81–9.
26 Lee S, Hwang J-I, Kim Y, Yoon PW, Ahn J, Yoo JJ. Venous Thromboembolism Following Hip and Knee Replacement Arthroplasty in Korea: A Nationwide Study Based on Claims Registry. *J Korean Med Sci* 2016; **31**: 80.
27 Pedersen AB, Sorensen HT, Mehnert F, Overgaard S, Johnsen SP. Risk Factors for Venous Thromboembolism in Patients Undergoing Total Hip Replacement and Receiving Routine Thromboprophylaxis. *J Bone Jt Surgery-American Vol* 2010; **92**: 2156–64.
28 Wallace G, Judge A, Prieto-Alhambra D, de Vries F, Arden NKNK, Cooper C. The effect of body mass index on the risk of post-operative complications during the 6 months following total hip replacement or total knee replacement surgery. *Osteoarthr Cartil* 2014; **22**: 918–27.
29 Pedersen AB, Mehnert F, Sorensen HT, Emmeluth C, Overgaard S, Johnsen SP. The risk of venous thromboembolism, myocardial infarction, stroke, major bleeding and death in patients undergoing total hip and knee replacement. *Bone Joint J* 2014; **96**-**B**: 479–85.
30 Belmont PJ, Goodman GP, Kusnezov NA, *et al.* Postoperative Myocardial Infarction and Cardiac Arrest Following Primary Total Knee and Hip Arthroplasty: Rates, Risk Factors, and Time of Occurrence. *J Bone Jt Surgery-American Vol* 2014; **96**: 2025–31.
31 Friedman R, Homering M, Holberg G, Berkowitz SD. Allogeneic Blood Transfusions and Postoperative Infections After Total Hip or Knee Arthroplasty. *J Bone Jt Surg* 2014; **96**: 272–8.

For peer review only - http://bmjopen.bmj.com/site/about/guidelines.xhtml

32 Illingworth KD, El Bitar YF, Banerjee D, Scaife SL, Saleh KJ. Inpatient Mortality After Primary Total Hip Arthroplasty: Analysis from the National Inpatient Sample Database. *J Arthroplasty* 2015; **30**: 369–73.
33 American Society of Anesthesiologists (ASA). ASA Physical Status Classification System. 2014. https://www.asahq.org/standards-and-guidelines/asa-physical-status-classification-system (accessed Feb 20, 2019).
34 Ferguson RJ, Silman AJ, Combescure C, *et al.* ASA class is associated with early revision and reoperation after total hip arthroplasty: an analysis of the Geneva and Swedish Hip Arthroplasty Registries. *Acta Orthop* 2019; : 1–13.

Acknowledgements

We acknowledge Professor Andy Judge for advice on statistical analysis at the earliest stages of this project . We are also grateful to Dr Andrew Clegg, and Stephen Pye for guidance in the use of the electronic frailty index. Dr John Griffiths and Professor Nigel Arden, provided very helpful advice at the design stage on the challenges of anaesthetic assessment and on studying osteoarthritis from an epidemiological perspective.

For peer review only

Figure: Flow chart of participants

For peer review only

Table 1: Demographic characteristics of cohort

**Gender**	N (%)
Female	4,090 (61.2)
**Age**	
65-69	1,294 (19.4)
70-74	1,976 (29.6)
75-79	1,736 (26.0)
80-84	1,095 (16.4)
85-89	465 (7.0)
>90	116 (1.7)
**Body Mass Index (BMI)**	
Underweight (<18.5)	54 (1.0)
Normal: (18.5-24.9)	1,615 (28.8)
Overweight: (25-29.9)	2,444 (43.6)
Obese: (>30)	1,497 (26.7)
Missing	1072
**Index of multiple deprivation**	
1 (Affluent)	1076 (25.5)
2	1718 (25.7)
3	1539 (23.0)
4	1144 (17.1)
5 (Deprived)	573 (8.6)
Missing	2

For peer review only

Table 2: Baseline data on measures of pre-operative health status (multimorbidity, frailty and morbidity burden)

**Charlson comorbidity index**	
0	4,490 (67.2)
1	682 (10.2)
2	880 (13.2)
3+	630 (9.4)
**Electronic frailty index (eFI)**	
0-4	4184 (62.6)
5-8	2,064 (30.9)
9-12	393 (5.9)
13+	41 (0.6)
**No. comorbid diseases**	
0	1,901 (28.5)
1	2,286 (34.2)
2	1,430 (21.4)
3+	1,065 (15.9)
**No. medications prescribed**	
0-4	1,963 (29.4)
5-7	1,832 (27.4)
8-12	1,992 (29.8)
13+	895 (13.4)
**No. contacts with primary care**	
0-7	2,167 (32.4)
8-11	1,598 (23.9)
12-17	1,528 (22.9)
18+	1,389 (20.8)

For peer review only

Table 3: Complications, length of stay, and readmission to hospital within 90 days

	N (%)
Any medical complication*	216 (3.2)
Death	57 (0.9)
Myocardial infarction	20 (0.3)
Venous thrombo-embolism	54 (0.8)
Stroke	8 (0.1)
Anaemia	12 (0.2)
Respiratory tract infection	28 (0.4)
Urinary tract infection	12 (0.2)
Wound infection	37 (0.6)
Length of stay (median, IQR)	6 (4-9)
Readmission to hospital	727 (10.9)

*Any medical complication includes death, myocardial infarction, venous thrombo-embolism, stroke, anaemia, respiratory tract infection, urinary tract infection, and wound infection

For peer review only

Table 4: Association between pre-operative morbidity status and 90-day medical complications

	No. at risk	Events	% Events	Unadjusted OR	Adjusted OR*
Charlson CI					
0	4490	120	2.67	Ref	Ref
1	682	27	3.96	1.50 (0.98, 2.30)	1.43 (0.93, 2.20)
2	880	43	4.89	1.87 (1.31, 2.67)	1.78 (1.24, 2.56)
3+	630	26	4.13	1.57 (1.02, 2.42)	1.46 (0.94, 2.26)
EFI					
0-4	4184	112	2.68	Ref	Ref
5-8	2064	75	3.63	1.37 (1.02, 1.85)	1.31 (0.96, 1.78)
9-12	393	26	6.62	2.58 (1.66, 4.00)	2.26 (1.42, 3.62)
13+	41	<5**			
Comorbid diseases					
0	1901	53	2.79	Ref	Ref
1	2286	53	2.32	0.83 (0.56, 1.22)	0.80 (0.54, 1.18)
2	1430	57	3.99	1.45 (0.99, 2.12)	1.41 (0.96, 2.08)
3+	1065	53	4.98	1.83 (1.24, 2.69)	1.74 (1.16, 2.61)
Prescriptions					
0-4	1963	49	3.09	Ref	Ref
5-7	1832	55	2.75	1.21 (0.82, 1.79)	1.17 (0.79, 1.73)
8-12	1992	68	3.53	1.38 (0.95, 2.00)	1.28 (0.88, 1.87)
13+	895	33	3.67	2.02 (1.33, 3.06)	1.85 (1.21, 2.83)
PC contacts					
0-7	2167	67	3.09	Ref	Ref
8-11	1598	44	2.75	0.89 (0.60, 1.31)	0.85 (0.58, 1.25)
12-17	1528	54	3.53	1.15 (0.80, 1.65)	1.09 (0.76, 1.58)
18+	1389	51	3.67	1.19 (0.82, 1.73)	1.07 (0.74, 1.57)
Total	6682	216			

*Adjusted for age, gender, region and calendar year of surgery

** The rules set for approval of use of CPRD data require that authors are not allowed to indicate the number of events in any category if they are less than 5

For peer review only - http://bmjopen.bmj.com/site/about/guidelines.xhtml

1 **Table 5: Association between pre-operative multimorbidity and 90-day mortality**

2

	No. at risk	Events	% Events	Unadjusted OR	Adjusted OR*
Charlson CI					
0	4490	32	0.71	Ref	Ref
1	682	6	0.88	1.24 (0.52, 2.97)	1.09 (0.45, 2.65)
2	880	13	1.48	2.09 (1.09, 4.00)	1.85 (0.95, 3.60)
3+	630	6	0.95	1.34 (0.56, 3.22)	1.12 (0.46, 2.73)
EFI					
0-4	4184	27	0.65	Ref	Ref
5-8	2064	20	0.97	1.51 (0.84, 2.69)	1.33 (0.72, 2.44)
9-12	393	10	2.54	4.02 (1.93, 8.37)	2.78 (1.24, 6.23)
13+	41	0	0	-	-
Comorbid diseases					
0	1901	14	0.74	Ref	Ref
1	2286	10	0.44	0.59 (0.26, 1.34)	0.58 (0.26, 1.33)
2	1430	16	1.12	1.53 (0.74, 3.14)	1.50 (0.71, 3.16)
3+	1065	17	1.60	2.19 (1.07, 4.45)	2.12 (0.99, 4.51)
Prescriptions					
0-4	1963	11	0.56	Ref	Ref
5-7	1832	19	1.04	1.86 (0.88, 3.92)	1.80 (0.85, 3.82)
8-12	1992	18	0.90	1.62 (0.76, 3.43)	1.39 (0.64, 2.98)
13+	895	9	1.01	1.80 (0.74, 4.37)	1.51 (0.61, 3.75)
Primary Care contacts					
0-7	2167	20	0.92	Ref	Ref
8-11	1598	13	0.81	0.88 (0.44, 1.78)	0.81 (0.40, 1.65)
12-17	1528	15	0.98	1.06 (0.54, 2.09)	0.90 (0.45, 1.78)
18+	1389	9	0.65	0.70 (0.32, 1.54)	0.53 (0.24, 1.19)
Total	6682	57			

3

4 *Adjusted for age, gender, region and calendar year of surgery

For peer review only

Table 6: Patient Reported Outcome Measures (PROMs)

	N	Mean (SD)
Oxford Hip Score		
Pre-operative	1460	17.9 (7.9)
Post-operative	1416	39.3 (8.2)
Change	1402	21.3 (9.7)
EuroQoL Index (EQ-5D)		
Pre-operative	1380	0.35 (0.31)
Post-operative	1367	0.80 (0.23)
Change	1285	0.45 (34)

For peer review only

Table 7: Post-operative Oxford Hip Score by multimorbidity

	No.	Pre-op	Post-op	Change	Unadjusted difference	Adjusted*
Charlson CI						
0	960	18.5 (8.0)	39.9 (8.0)	21.4 (9.6)	Ref	Ref
1	113	17.6 (8.4)	37.5 (9.7)	20.1 (10.5)	-2.0 (-3.6, -0.5)	-2.0 (-3.6, -0.5)
2	199	16.0 (7.5)	37.8 (8.3)	21.9 (9.4)	-1.4 (-2.6, -0.2)	-1.0 (-2.2, 0.2)
3+	130	16.8 (7.1)	38.5 (8.1)	21.5 (10.0)	-1.15 (-2.5, 0.4)	-0.60 (-2.0, 0.8)
EFI						
0-4	798	18.8 (7.9)	40.7 (7.5)	21.8 (9.4)	Ref	Ref
5-8	511	17.3 (7.9)	37.7 (8.8)	20.5 (10.2)	-2.5 (-3.4, -1.7)	-2.0 (-2.9, -1.2)
9-12	86	14.6 (7.0)	35.8 (8.1)	21.4 (8.8)	-3.6 (-5.4, -1.9)	-2.9 (-4.7, -1.1)
13+	7	14.7 (8.2)	39.1 (10.4)	24.4 (8.7)	-0.45 (-6.2, 5.3)	1.0 (-4.8, 6.7)
Comorbid dis						
0	358	18.9 (7.8)	40.7 (7.9)	21.7 (9.9)	Ref	Ref
1	481	18.6 (8.1)	39.9 (7.9)	21.3 (9.6)	-0.7 (-1.8, 0.4)	-0.4 (-1.5, 0.6)
2	324	17.6 (7.9)	38.7 (8.2)	21.0 (9.6)	-1.6 (-2.8 , -0.4)	-1.2 (-2.3, 0.1)
3+	239	15.7 (7.5)	36.9 (8.9)	21.2 (9.7)	-3.0 (-4.3, -1.7)	-2.3 (-3.6, -1.0)
Prescriptions						
0-4	407	19.9 (7.4)	41.4 (7.1)	21.4 (9.3)	Ref	Ref
5-7	383	19.4 (8.1)	40.4 (7.4)	20.9 (9.5)	-0.8 (-1.9, 0.3)	-0.5 (-1.6, 0.6)
8-12	406	16.6 (7.7)	38.2 (8.5)	21.7 (9.9)	-2.3 (-3.4, -1.2)	-2.0 (-3.0, -0.9)
13+	206	14.1 (7.3)	35.4 (9.2)	21.3 (10.3)	-4.6 (-5.9, -3.2)	-4.0 (-5.3, -2.4)
PC contacts						
0-7	2167	18.9 (7.8)	39.9 (7.8)	21.0 (8.9)	Ref	Ref
8-11	1598	18.3 (8.1)	40.1 (7.8)	21.7 (10.0)	0.3 (-0.8 , 1.5)	0.6 (-0.6, 1.7)
12-17	1528	17.8 (7.8)	39.4 (8.1)	21.7 (9.9)	-0.1 (-1.2, 1.0)	0.4 (-0.7, 1.5)
18+	1389	16.1 (9.1)	37.2 (9.2)	21.1 (10.3)	-2.0 (-3.2, -0.8)	-1.5 (-2.7, -0.3)
Total	1402	1460	1416	1402		

*Adjusted for age, gender, region and calendar year of surgery

For peer review only

For peer review only - http://bmjopen.bmj.com/site/about/guidelines.xhtml

Table 8: Post-operative EQ5D by multimorbidity

	No.	Pre-op	Post-op	Change	Unadjusted difference	Adjusted difference*
Charlson CI						
0	880	.37 (.31)	.82 (.22)	.45 (.34)	Ref	Ref
1	108	.33 (.32)	.77 (.25)	.44 (.33)	-.036 (-.080, .007)	-.037 (-.080, .007)
2	178	.29 (.31)	.76 (.22)	.48 (.34)	-.038 (-.073, -.002)	-.028 (-.064, .007)
3+	119	.33 (.29)	.74 (.24)	.40 (.33)	-.072 (-.114, -.030)	-.060 (-.102, -.018)
EFI						
0-4	735	.39 (.31)	.83 (.20)	.45 (.33)	Ref	Ref
5-8	464	.32 (.31)	.76 (.23)	.45 (.36)	-.059 (-.084, -.033)	-.048 (-.073, -.022)
9-12	79	.20 (.29)	.69 (.28)	.47 (.34)	-.141 (-.192, -.090)	-.126 (-.178, -.074)
13+	7	.20 (.35)	.78 (.35)	.59 (.32)	-.021 (-.184, .141)	.004 (-.157, .166)
Comorbid dis						
0	330	.39 (.31)	.85 (.19)	.46 (.34)	Ref	Ref
1	429	.38 (.31)	.81 (.20)	.43 (.32)	-.037 (-.068, -.005)	-.030 (-.062, .001)
2	306	.33 (.31)	.78 (.23)	.45 (.35)	-.060 (-.094, -.026)	-.049 (-.084, -.015)
3+	220	.26 (.32)	.71 (.28)	.45 (.36)	-.122 (-.159, -.084)	-.105 (-.143, -.067)
Prescriptions						
0-4	378	.42 (.30)	.85 (.20)	.44 (.34)	Ref	Ref
5-7	351	.41 (.30)	.83 (.20)	.42 (.32)	-.023 (-.055, .008)	-.062 (-.062, .001)
8-12	372	.30 (.30)	.77 (.22)	.47 (.34)	-.071 (-.103, -.040)	-.084 (-.084, -.015)
13+	184	.19 (.31)	.69 (.28)	.50 (.37)	-.139 (-.178, -.099)	-.143 (-.143, -.067)
PC contacts						
0-7	2167	.39 (.31)	.82 (.22)	.44 (.33)	Ref	Ref
8-11	1598	.36 (.30)	.83 (.21)	.46 (.34)	.003 (-.029, .036)	.007 (-.025, .039)
12-17	1528	.36 (.31)	.80 (.21)	.44 (.35)	-.018 (-.049, .013)	-.010 (-.042, .021)
18+	1389	.26 (.32)	.72 (.26)	.47 (.35)	-.079 (-.114, -.044)	-.070 (-.106, -.034)
Total	1285	1380	1367	1285		

*Adjusted for age, gender, region and calendar year of surgery

For peer review only

1

For peer review only

For peer review only - http://bmjopen.bmj.com/site/about/guidelines.xhtml

Figure: Flow chart of available data

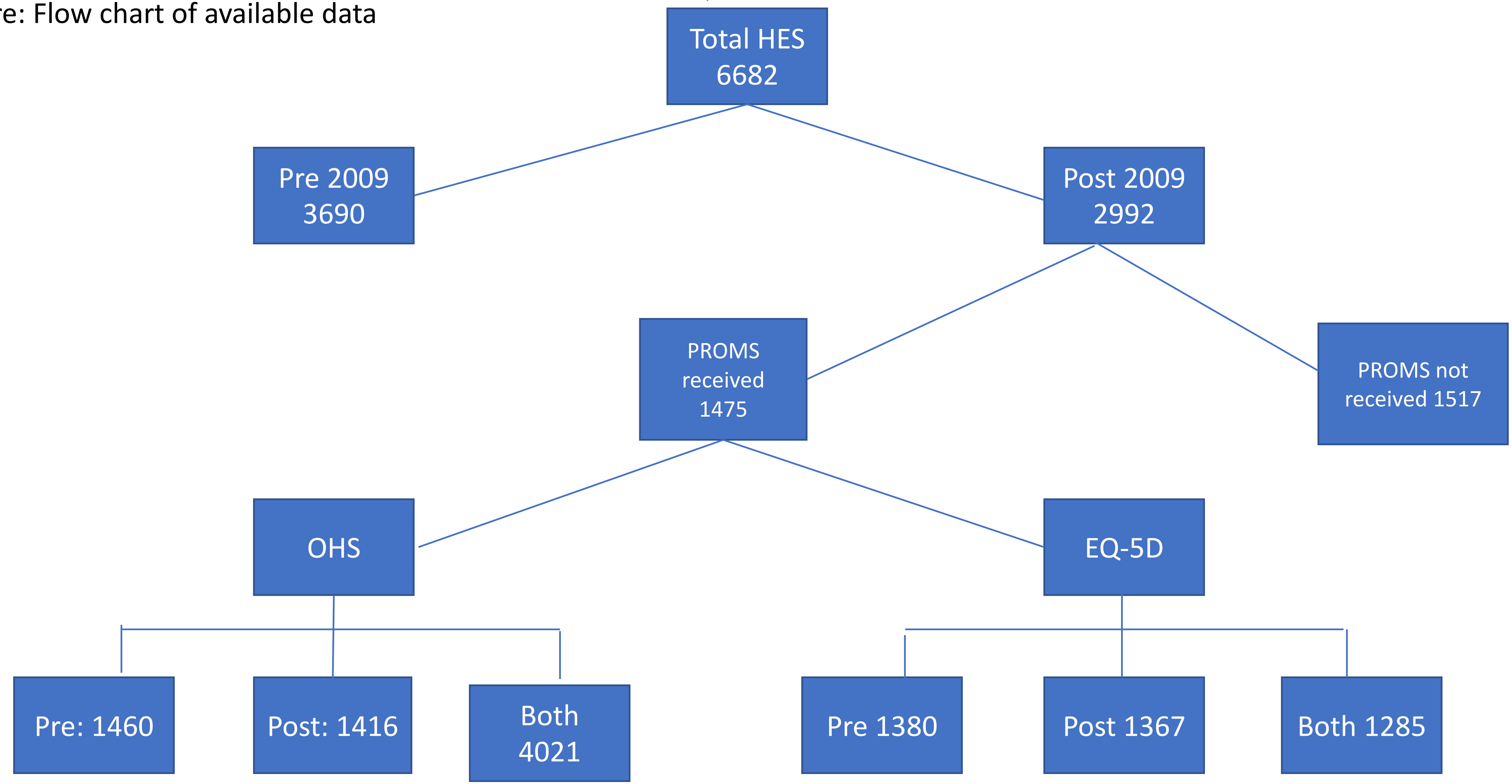

Appendix: Interpretation of Oxford Hip Scores and EQ-5D in patients receiving a hip arthroplasty

The OHS is a 12-item score with 4 levels, thus scores vary between 0-48, with higher scores being the best outcome. The clinically meaningful difference is considered to be an increase of +11 points and a change of +5 is considered statistically meaningful (ie greater than background 'noise')

Beard DJ, Harris K, Dawson J, *et al.* Meaningful changes for the Oxford hip and knee scores after joint replacement surgery. *J Clin Epidemiol* 2015; **68**: 73–9.

The EQ-5D has been very widely studied in several disease areas. There have been several studies looking at what is a clinically meaningful difference and a meta-analysis yielded an average difference of 0.18

Coretti S, Ruggeri M, McNamee P. The minimum clinically important difference for EQ-5D index: A critical review. Expert Rev. Pharmacoeconomics Outcomes Res. 2014; **14**: 221–33

For peer review only

Supplementary Table 1: Baseline data on measures of pre-operative health status (multimorbidity, frailty and morbidity burden) in patients who underwent primary THR in HES APC during the date period in which PROMs were collected (N=2992)

	PROMs data	No PROMs data
**Charlson comorbidity index**		
0	1,013 (68.7)	931 (61.4)
1	123 (8.3)	152 (10.0)
2	205 (13.9)	254 (16.7)
3+	134 (9.1)	180 (11.9)
**Electronic frailty index (eFI)**		
0-4	831 (56.3)	752 (49.6)
5-8	544 (36.9)	593 (39.1)
9-12	93 (6.3)	151 (10.0)
13+	7 (0.5)	21 (1.4)
**No. comorbid diseases**		
0	378 (25.6)	320 (21.1)
1	497 (33.7)	464 (30.6)
2	346 (23.5)	364 (24.0)
3+	254 (17.2)	369 (24.3)
**No. medications prescribed**		
0-4	428 (29.0)	400 (26.4)
5-7	401 (27.2)	375 (24.7)
8-12	429 (29.1)	500 (33.0)
13+	217 (14.7)	242 (16.0)
**No. contacts with primary care**		
0-7	471 (31.9)	464 (30.6)
8-11	335 (22.7)	358 (23.6)
12-17	384 (26.0)	334 (22.0)
18+	285 (19.3)	361 (23.8)
**Total**	1475	1517

For peer review only

Supplementary Table 2

Influence of pre-operative health status on risk of hospital readmission within 90 days

	No. patients	Events	% Events	Unadjusted OR (95% CI)	Adjusted OR* (95% CI)
Charlson Comorbidity Index					
0	4490	477	10.6	Ref	Ref
1	682	82	12.0	1.15 (0.90, 1.48)	1.09 (0.85, 1.40)
2	880	131	14.9	1.47 (1.19, 1.81)	1.41 (1.14, 1.74)
3+	630	96	15.2	1.51 (1.19, 1.92)	1.45 (1.14, 1.84)
Count of chronic diseases					
0	1901	192	10.1	Ref	Ref
1	2286	232	10.2	1.01 (0.82, 1.23)	1.01 (0.82, 1.23)
2	1430	205	14.3	1.49 (1.21, 1.84)	1.47 (1.19, 1.83)
3+	1065	157	14.7	1.54 (1.23, 1.93)	1.53 (1.21, 1.94)
Count of medications prescribed					
0-4	1963	180	9.2	Ref	Ref
5-7	1832	201	11.0	1.22 (0.99, 1.51)	1.22 (0.99, 1.51)
8-12	1992	257	12.9	1.47 (1.20, 1.80)	1.45 (1.18, 1.78)
13+	895	148	16.5	1.96 (1.55, 2.48)	1.96 (1.54, 2.49)
Count of contacts with primary care					
0-7	2167	204	9.4	Ref	Ref
8-11	1598	172	10.8	1.16 (0.94, 1.44)	1.15 (0.93, 1.43)
12-17	1528	192	12.6	1.38 (1.12, 1.70)	1.37 (1.11, 1.69)
18+	1389	218	15.7	1.79 (1.46, 2.20)	1.75 (1.42, 2.15)
Electronic Frailty Index					
0-4	4184	432	10.3	Ref	Ref
5-8	2064	270	13.1	1.31 (1.11, 1.54)	1.30 (1.10, 1.55)
9-12	393	76	19.3	2.08 (1.59, 2.73)	2.01 (1.51, 2.67)
13+	41	8	19.5	2.11 (0.97, 4.59)	2.15 (0.97, 4.73)
Total	6682	786	11.8		

*The model was adjusted for age, sex, region of the residence, and year of surgery.

For peer review only

Supplementary Table 3:

Influence of pre-operative health status on length of stay in hospital

	Total at risk	Median length of stay (IQR)
Charlson Comorbidity Index		
0	4490	6 (4, 9)
1	682	6 (5, 9)
2	880	6 (4, 9)
3+	630	7 (5, 9)
Count of chronic diseases		
0	1901	6 (4, 8)
1	2286	6 (4, 9)
2	1430	6 (4, 9)
3+	1065	7 (5, 10)
Count of medications prescribed		
0-4	1963	6 (4, 8)
5-7	1832	6 (4, 9)
8-12	1992	7 (5, 9)
13+	895	7 (5, 10)
Count of contacts with primary care		
0-7	2167	6 (4, 8)
8-11	1598	6 (4, 9)
12-17	1528	6 (4, 9)
18+	1389	7 (5, 10)
Electronic Frailty Index		
0-4	4184	6 (4, 9)
5-8	2064	6 (4, 9)
9-12	393	8 (5, 11)
13+	41	8 (7, 13)
Total	6682	6 (4, 9)

| ,

For peer review only

For peer review only

Reporting checklist for cohort study.

Based on the STROBE cohort guidelines.

Instructions to authors

Complete this checklist by entering the page numbers from your manuscript where readers will find each of the items listed below.

Your article may not currently address all the items on the checklist. Please modify your text to include the missing information. If you are certain that an item does not apply, please write "n/a" and provide a short explanation.

Upload your completed checklist as an extra file when you submit to a journal.

In your methods section, say that you used the STROBE cohortreporting guidelines, and cite them as:

von Elm E, Altman DG, Egger M, Pocock SJ, Gotzsche PC, Vandenbroucke JP. The Strengthening the Reporting of Observational Studies in Epidemiology (STROBE) Statement: guidelines for reporting observational studies.

		Reporting Item	Page Number (lines)
**Title and abstract**			
Title	#1a	Indicate the study's design with a commonly used term in the title or the abstract	1
Abstract	#1b	Provide in the abstract an informative and balanced summary of what was done and what was found	3
**Introduction**			
Background / rationale	#2	Explain the scientific background and rationale for the investigation being reported	5
Objectives	#3	State specific objectives, including any prespecified hypotheses	5 (22-25)
**Methods**			
Study design	#4	Present key elements of study design early in the paper	5 (34-37)

For peer review only

Setting	#5	Describe the setting, locations, and relevant dates, including periods of recruitment, exposure, follow-up, and data collection	6 (5-14)
Eligibility criteria	#6a	Give the eligibility criteria, and the sources and methods of selection of participants. Describe methods of follow-up.	6 (17-22)
Eligibility criteria	#6b	For matched studies, give matching criteria and number of exposed and unexposed	NA
Variables	#7	Clearly define all outcomes, exposures, predictors, potential confounders, and effect modifiers. Give diagnostic criteria, if applicable	6 (24)-7 (3)
Data sources / measurement	#8	For each variable of interest give sources of data and details of methods of assessment (measurement). Describe comparability of assessment methods if there is more than one group. Give information separately for for exposed and unexposed groups if applicable.	6 (17) – 7 (3)
Bias	#9	Describe any efforts to address potential sources of bias	8 (5-9)
Study size	#10	Explain how the study size was arrived at	7 (27-30)
Quantitative variables	#11	Explain how quantitative variables were handled in the analyses. If applicable, describe which groupings were chosen, and why	7 (6-14)
Statistical methods	#12a	Describe all statistical methods, including those used to control for confounding	7 (17-22)
Statistical methods	#12b	Describe any methods used to examine subgroups and interactions	NA
Statistical methods	#12c	Explain how missing data were addressed	8 (1-9)
Statistical methods	#12d	If applicable, explain how loss to follow-up was addressed	NA
Statistical methods	#12e	Describe any sensitivity analyses	8 (5-9)
**Results**			
Participants	#13a	Report numbers of individuals at each stage of study—eg numbers potentially eligible, examined for eligibility, confirmed eligible,	Figure

For peer review only

		included in the study, completing follow-up, and analysed. Give information separately for for exposed and unexposed groups if applicable.	
Participants	#13b	Give reasons for non-participation at each stage	NA
Participants	#13c	Consider use of a flow diagram	Figure
Descriptive data	#14a	Give characteristics of study participants (eg demographic, clinical, social) and information on exposures and potential confounders. Give information separately for exposed and unexposed groups if applicable.	Table 1
Descriptive data	#14b	Indicate number of participants with missing data for each variable of interest	Table 1
Descriptive data	#14c	Summarise follow-up time (eg, average and total amount)	6 (35-41)
Outcome data	#15	Report numbers of outcome events or summary measures over time. Give information separately for exposed and unexposed groups if applicable.	Tables 3,6
Main results	#16a	Give unadjusted estimates and, if applicable, confounder-adjusted estimates and their precision (eg, 95% confidence interval). Make clear which confounders were adjusted for and why they were included	Tables 4,5,7,8
Main results	#16b	Report category boundaries when continuous variables were categorized	Table 2
Main results	#16c	If relevant, consider translating estimates of relative risk into absolute risk for a meaningful time period	NA
Other analyses	#17	Report other analyses done—e.g., analyses of subgroups and interactions, and sensitivity analyses	Supp Table 1
**Discussion**			
Key results	#18	Summarise key results with reference to study objectives	9
Limitations	#19	Discuss limitations of the study, taking into account sources of potential bias or imprecision. Discuss both direction and magnitude of any potential bias.	9-11

For peer review only

Interpretation	#20	Give a cautious overall interpretation considering objectives, limitations, multiplicity of analyses, results from similar studies, and other relevant evidence.	11
Generalisability	#21	Discuss the generalisability (external validity) of the study results	11
**Other Information**			
Funding	#22	Give the source of funding and the role of the funders for the present study and, if applicable, for the original study on which the present article is based	2

The STROBE checklist is distributed under the terms of the Creative Commons Attribution License CC-BY. This checklist was completed on 20. October 2020 using https://www.goodreports.org/, a tool made by the EQUATOR Network in collaboration with Penelope.ai

For peer review only

BMJ Open

Does pre-existing morbidity influence risks and benefits of total hip replacement for osteoarthritis: a prospective study of 6682 patients from linked national datasets in England?

Journal:	*BMJ Open*
Manuscript ID	bmjopen-2020-046712.R2
Article Type:	Original research
Date Submitted by the Author:	14-Jun-2021
Complete List of Authors:	Ferguson, Rory; University of Oxford Nuffield Department of Orthopaedics Rheumatology and Musculoskeletal Sciences, Botnar Institute<br>Prieto-Alhambra, Daniel; University of Oxford, Centre for Statistics in Medicine, NDORMS<br>Peat, George; Keele University, Research Institute for Primary Care & Health Sciences<br>Delmestri, Antonella; University of Oxford, Nuffield Department of Orthopaedics, Rheumatology, and Musculoskeletal Sciences<br>Jordan, Kelvin; Keele University, School of Primary, Community and Social Care<br>Strauss, Vicky; University of Oxford, Centre for Statistics in Medicine, Ndorms<br>Valderas, Jose; University of Exeter Medical School,<br>Walker, Christine; Keele University, Research User Group, Arthritis Research UK Primary Care Centre, Research Institute of Primary Care and Health Sciences<br>Yu, Dahai; Keele University, Research Institute for Primary Care & Health Sciences<br>Glyn-Jones, Sion; University of Oxford, NDORMS<br>Silman, Alan; University of Oxford Nuffield Department of Orthopaedics Rheumatology and Musculoskeletal Sciences, Botnar Institute
**Primary Subject Heading**:	Surgery
Secondary Subject Heading:	Geriatric medicine, Rheumatology
Keywords:	GERIATRIC MEDICINE, SURGERY, Hip < ORTHOPAEDIC & TRAUMA SURGERY, ORTHOPAEDIC & TRAUMA SURGERY

SCHOLARONE™
Manuscripts

I, the Submitting Author has the right to grant and does grant on behalf of all authors of the Work (as defined in the below author licence), an exclusive licence and/or a non-exclusive licence for contributions from authors who are: i) UK Crown employees; ii) where BMJ has agreed a CC-BY licence shall apply, and/or iii) in accordance with the terms applicable for US Federal Government officers or employees acting as part of their official duties; on a worldwide, perpetual, irrevocable, royalty-free basis to BMJ Publishing Group Ltd ("BMJ") its licensees and where the relevant Journal is co-owned by BMJ to the co-owners of the Journal, to publish the Work in this journal and any other BMJ products and to exploit all rights, as set out in our licence.

The Submitting Author accepts and understands that any supply made under these terms is made by BMJ to the Submitting Author unless you are acting as an employee on behalf of your employer or a postgraduate student of an affiliated institution which is paying any applicable article publishing charge ("APC") for Open Access articles. Where the Submitting Author wishes to make the Work available on an Open Access basis (and intends to pay the relevant APC), the terms of reuse of such Open Access shall be governed by a Creative Commons licence – details of these licences and which Creative Commons licence will apply to this Work are set out in our licence referred to above.

Other than as permitted in any relevant BMJ Author's Self Archiving Policies, I confirm this Work has not been accepted for publication elsewhere, is not being considered for publication elsewhere and does not duplicate material already published. I confirm all authors consent to publication of this Work and authorise the granting of this licence.

For peer review only

Does pre-existing morbidity influence risks and benefits of total hip replacement for osteoarthritis: a prospective study of 6682 patients from linked national datasets in England

RJ Ferguson [1]
D Prieto-Alhambra [1]
G Peat [2]
A Delmestri [1]
KP Jordan [2]
V Strauss [1]
JM Valderas [3]
C Walker [2]
D Yu[2]
S Glyn-Jones [1,4]
AJ Silman[1]

[1]Nuffield Department of Orthopaedics, Rheumatology and Musculoskeletal Sciences, University of Oxford, Oxford, UK.
[2]Primary Care and Health Sciences Department, Keele University, Keele, UK.
[3]University of Exeter Medical School, Exeter, UK.
[4]Oxford University Hospital NHS Foundation Trust, Oxford, UK.

Correspondence to Professor Silman
Nuffield Department of Orthopaedics, Rheumatology and Musculoskeletal Sciences,
University of Oxford
Botnar Research Centre, Windmill Road
Headington, Oxford, OX3 7LD.
alan.silman@ndorms.ox.ac.uk

Word count abstract, main paper 3320

Key words: Hip osteoarthritis, arthroplasty, multimorbidity, mortality, outcome, quality of life

Ethical Approval

No ethical approval was required and anonymised data were provided by as above. No individual patient consent was therefore required.

Consent for publication: not applicable

For peer review only

Acknowledgements

We acknowledge Professor Andy Judge for advice on statistical analysis at the earliest stages of this project . We are also grateful to Professor Andrew Clegg, and Stephen Pye for guidance in the use of the electronic frailty index. Dr John Griffiths and Professor Nigel Arden, provided very helpful advice at the design stage on the challenges of anaesthetic assessment and on studying osteoarthritis from an epidemiological perspective. We are grateful to public members of the Research Users Group at Keele University for their helpful input especially in the interpretation of the results

For peer review only

Abstract

Total hip arthroplasty (THA) surgery for elderly people with multimorbidity increases the risk of serious health hazards including mortality. Whether such background morbidity reduces the clinical benefit is less clear

Objective

To evaluate how pre-existing health status, using multiple approaches, influences risks of, and quality of life benefits from, THA.

Setting

Longitudinal record linkage study of a UK sample linking their primary care to their secondary care records

Participants

All 6682 patients, between 1995 and 2014, with hip osteoarthritis identified from the national primary care register who had a THA. Data from their primary care record was extracted to score 5 different constructs: Charlson Comorbidity Index, Electronic Frailty Index (eFI) and counts of comorbidity disorders (from list of 17), prescribed medications and number of primary care visits

Outcome measures

Linked secondary care register provided data on post-operative mortality and major medical complications. Linkage to the national Patients Reported Outcome Measures (PROMs) database permitted assessment of change in hip-specific and general improvement in quality of life using Oxford Hip Score (OHS) and EQ-5D respectively

Results

Peri-operative complication rate was 3.2% and mortality was 0.9%, both increased with worse pre-operative health status although this relationship varied depending on the morbidity construct: the eFI showing the strongest relationship but number of visits having no predictive value. By contrast, the benefits were not reduced in those with worse pre-operative health however assessed: improvement with both OHS or EQ-5D, was observed in all the morbidity strata.

Conclusions

Independent of pre-operative morbidity, THA leads to similar substantial improvements in quality of life. These are offset by an increase in medical complications in some subgroups of patients with high morbidity, depending on the definition used. For most elderly people, their other health disorders should not be a barrier for THA.

(293 words)

For peer review only

Strengths and limitation

- National large sample of older patients recorded in primary care with newly reported osteoarthritis of the hip and who had received a hip replacement
- Multi-dimensional approach to assessing their concurrent morbidity and health status prior to surgery from the primary care record
- Linkage to their surgical outcomes both hazards - mortality and significant post-operative complications and the benefits - improvement in quality of life
- Such study using routine national datasets have advantage of size, access to complete health record and linkage to both data hazards and benefits from surgery
- Relying on routine data has the challenges of completeness of recording and accuracy of the data items extracted

For peer review only

Background

National data on the short term outcome from total hip arthroplasty (THA) demonstrate a substantial improvement in quality of life[1]. Most recipients are older adults: the median age for example in the UK countries reporting to the National Joint Registry is 69.[2]

With increasing age, patients are increasingly likely to develop multiple chronic health disorders (multimorbidity) with many patients described as frail. [3–7] Such disorders increase the hazards from surgery[8-10] and may also limit the benefits in quality of life.[11-14] There is in practice an inevitable selection process prior to surgical acceptance of older adults with multimorbidity, though there is no specific guidance on how these factors should influence surgical decision making, either in UK [15] or USA [16]:

There are challenges in identifying a valid approach to measure the cumulative severity of all chronic health disorders.[17] Simple counts of the presence of specific chronic diseases, or weighted instruments (eg Charlson score[18]), typically focus on disorders associated with the hazards especially mortality but exclude those that might impact on quality of life[19]. In a companion paper to this one we have demonstrated that in current practice in the UK, pre-existing health problems, even when only moderate in effect, do influence the likelihood and timing of THA. The question then arose is whether this impact on acceptance for surgery is appropriately reflects how far these health issues impact on surgical outcome [20].

Our goal was to identify the impact that multimorbidity and frailty on the risks and benefits of THA. Our specific objectives were to assess, using a number of different approaches to scoring, how multimorbidity influenced the risks and benefits of elective total hip replacement for osteoarthritis. The former outcome was assessed by post-operative complications, length of stay and hospital readmission; and the latter assessed by patient reported outcome scores (PROMs) covering pain, function and quality of life. To maximise the external validity, the study took advantage of the availability of English national datasets linking primary and secondary care.

Methods

Summary of design

In a longitudinal record linkage study using a national database, patients with a newly recorded diagnosis of hip osteoarthritis were identified from their primary care record. Linkage to the national secondary care database identified those individuals who had a THA, who became the subjects of the study. The primary care record was interrogated to provide information on other health disorders and treatments were used to derive measures of pre-existing multimorbidity and health status at the time of surgery. This secondary care database also provided data on peri-operative death and major complications as well as scores of patient outcome. The influence of the scores from these measures on mortality, in-

For peer review only - http://bmjopen.bmj.com/site/about/guidelines.xhtml

patient complications and readmission within 90 days and PROMs at 6 months were then calculated.

Data sources

We used the UK Clinical Practice Research Datalink (CPRD) which contains the primary care electronic medical records of approximately 4.4 million active patients and representative of the wider UK population.[21] Data are stored with Read codes for diseases that are cross-referenced to the International Classification of Diseases (ICD-10).

The Hospital Episode Statistics dataset (HES) contains all patient attendances at NHS hospitals in England, and covers episodes of care. HES records patient demographic data, diagnoses and procedures and is linked to national death certificate data and these data were provided anonymised. HES has also been linked since 2009 to the PROMs database gathering outcome data NHS-funded THA both pre-operatively and at 6 months post-operatively.[22]

Study Population

All patients in the CPRD with a READ code for hip osteoarthritis from January 1 1995, until March 31, 2014, and aged 65 years or over at the time of diagnosis, were identified (ISAC protocol number 17_024R). The validation of those Read codes for hip osteoarthritis are described elsewhere.[23] Those patients who underwent elective primary THA, identified from HES were included in the analysis. Operations were identified from the Classification of Surgical Operations and Procedures (OPCS-4) codes.

The following measures of multimorbidity prior to the date of surgery were extracted from the accumulated primary care record. These were: (i) Charlson Comorbidity Index (CCI)-developed to predict 1-year mortality based on the presence of specified chronic diseases.[18], (ii) count of the 17 chronic diseases listed in the NHS Quality Outcomes Framework (QOF).[24] (iii) electronic Frailty Index (eFI), a score based on the cumulative deficit model of frailty and validated against mortality, hospitalisation and nursing home admission.[25] We also used 2 measures of the burden of care associated with the presence of chronic conditions. Thus, within the 12 months prior to surgery we extracted the numbers of (i) different prescribed medications and (ii) primary care contacts for any reason.

Outcomes

All subjects had the following outcomes, in addition to death, extracted from the linked HES data within 90 days of THA: venous thromboembolism, myocardial infarction, stroke, anaemia, lower respiratory tract infection, urinary tract infection, wound infection. Also extracted were length of stay following surgery and readmission after discharge within 90 days. The national PROMs database provided data since 2009 on (i) Oxford Hip Score as a measure of hip function and (ii) EQ-5D Index as a measure of quality of life at 6 months post-operatively on all patients receiving a THA – information on the appropriate interpretation of

For peer review only

these scores is provided in the Appendix. Although all patients are invited to complete the PROMs questionnaires pre-operatively and at 6 months post-operatively, completion rates vary. Further some respondents delay completing their post-operative scores beyond the 6 months.[22]

Statistical analysis

The scores for each of the five measures of multimorbidity were split into 4 categories. The eFI has 4 categories defined by the developers: fit (0-4), mild frailty (5-8), moderate frailty (9-12), and severe frailty (13+). The actual CCI scores were used to split into categories of score of 0, 1, 2, and 3+. Based on the distribution of the data the counts of number of chronic diseases were also split in categories of score of 0, 1, 2, and 3+. The categories for the number of medications prescribed and of primary care visits were derived from the observed distributions of the actual data, aiming for equal sized quarters: with medication counts divided into scores of 0-4, 5-7, 8-12, and 13+ and number of primary care visits into 0-7, 8-11, 12-17 and 18+. The lowest category for each score was used as the referent category in the analysis.

Logistic regression methods were used to calculate the unadjusted odds of a complication or readmission to hospital by multimorbidity category. Poisson regression methods were used to calculate the unadjusted difference in length of stay by multimorbidity category. Linear regression methods were used to calculate the difference in post-operative OHS and EQ-5D score, adjusted for pre-operative score, by multimorbidity category. All models were then adjusted for age at surgery, sex, region of the UK, and calendar year of total hip replacement.

Patient and public Involvement

This research was funded by the NIHR Research for Patient Benefit scheme which has an absolute requirement that there is patient and public involvement in all relevant stages of the research. The research question had been originally raised in a "Priority Setting Partnership for Priorities for Research in Hip and Knee Arthroplasty" and this was followed by a survey of members of the Keele PPI panel: Research Users Group (RUG). We then tested the suggested questions with the group and received very positive feedback. One of the RUG members (Author CW), herself a patient with direct experience of the target of the research, then became an active member of the research team, participated in all the meetings and advised on the design including the questions that should be asked during the analysis stage. As part of the dissemination phase, Keele PPI group organised a round table event attended by the lead authors where the results were discussed and guidance given on how those members of the public who were present wished to see disseminated.

Results

For peer review only - http://bmjopen.bmj.com/site/about/guidelines.xhtml

Baseline data

The demographic characteristics of the study participants are shown in Table 1. There were 6682 patients with a code for hip osteoarthritis on the primary care record who after linkage to the HES data, had had a THA within the follow up period from April 1997 to March 2014. The mean age at surgery was 76 years (SD 6).

Pre-operative scores for the four multimorbidity measures derived are shown in Table 2. Only 20% had a CCI above 1 perhaps reflecting the selection of patients for surgery. Based on the eFI classification, 34% were classified as 'mildly frail' and 6.5% 'moderately frail'. Around 1 in 6 had 3 or more chronic disorders recorded. Over 40% had been prescribed at least 8 medications, and a similar proportion had more than 12 primary care visits, in the previous 12 months.

For peer review only

Outcomes

Data on complications, length of stay and readmission to hospital were available on all patients (as linkage to HES was a criterion for study entry). As mentioned above, data on PROMs was only available on a subset of patients who completed the questionnaires (Figure). In total, pre- and post-operative OHS was available on 1402 patients, and pre- and post-operative EQ-5D Index was available on 1285 patients. As a test of bias, we compared the multimorbidity measures between those with and without PROMs (Supplementary Table 1) which whilst showing no substantive shifts, across all the measures, those who did not provide PROMS were more likely to have worse multimorbidity and also reside in more socio economically deprived areas.

Association between multimorbidity measures and adverse outcomes

In all, 216 (3.2%) patients suffered a post-operative complication including thrombo-embolism, myocardial infarction, urine and respiratory infections and wound infection, and 57 (0.9%) patients died, within 90 days of surgery (Table 3). The rate of all-cause readmission to hospital within 90 days of surgery was 11%.

The impact of multimorbidity scores across all these medical complications are shown in Table 4. The overall adverse event rate was only modestly increased with increasing levels of multimorbidity relative to the lowest scoring group. These increases in risks were attenuated after adjustment for age and the other possible confounders. Those in highest frailty group were the most predictive of a medical complication. By contrast the other measures of multimorbidity were only weakly predictive; with some measures such as the number of primary care visits showing no trend We analysed the individual complications separately (data not shown). The numbers with any of the individual complications were small but there were no substantial difference between the risks of individual complications in their relationship with the multimorbidity scores.

The data on mortality are shown in Table 5. The mortality risk in the least healthy stratum ranged from 0.7% for the group with the highest number of primary care contacts to 2.3% for those with an eFI of 9 or more. There was an inconsistent association between death and Charlson score but the number of events in those with a Charlson score above 2 were too small for useful analysis. Neither the groups with the number of prescribed medications nor of primary care contacts predicted mortality. The measure with the greatest predictive power of death was the eFI. After adjustment for age and gender, those in the two highest frailty groups had a 2.5 fold increased mortality risk. The overall number of deaths though was modest and the resulting small numbers when stratified by pre-operative morbidity category, limits the precision of these estimates.

The impact of multimorbidity scores on length of stay were also analysed. Detailed data are not shown but in brief for all the multimorbidity measures, apart from the eFI, there was no impact on length of stay. The median length of stays in the lowest and highest categories of multimorbidity were: 6 and 7 days for Charlson score, 6 and 8 days for eFI score, 6 and 7 days for the count of chronic diseases, and 6 and 7 days for the count of medications prescribed.

We have also examined the impact of these pre-operative health measures on 2 indirect measures of complications: readmission rates and length of stay (LOS). Overall there were 786 patients readmitted for any reason over the next 90 days. Risks were related to level of pre-operative health with those in the worst health categories having a twofold increased risk compared to those in in the healthiest group. The results were broadly similar independent of the scoring approach (Supplementary Table 2). As regards LOS, the differences between grades of prior ill health were modest, with the median and interquartile ranges almost identical. The eFI was a slight outlier where those in the highest two categories had a median LOS two days higher than those with lesser frailty (Supplementary Table 3).

Association between multimorbidity measures and quality of life

Across all patients, there was an overall marked improvement in both the specific (OHS) and the generic (EQ-5D) patient outcomes. The summary outcome data are shown in Table 6, confirming substantial improvement at 6 months in both the OHS and EQ-5D across the population studied. The mean improvement in OHS of around 20 did not vary importantly between the different levels of any of the approaches to assessing the pre-operative morbidity (Table 7). For each of our measures of multimorbidity, the pre-operative OHS showed was lower with increasing score. What was of note was the similarity in the relative improvement in the post -operative OHS seen across the board for all measures of multimorbidity. Typically mean pre-operative scores in the mid to late teens reached scores

For peer review only

in the high 30's and 40's: the highest score achievable is 48. Thus, surgery led to a marked improvement, not attenuated by the severity of the pre-operative general health.

A similar analysis was undertaken on EQ5D (Table 8). As expected, those with a higher pre-operative morbidity had a lower pre-operative EQ5D, reflecting the impact of the patients' other comorbid disorders on their overall quality of life. However, the 6 month incremental improvement in EQ5D was almost identical across all categories of multimorbidity, independent of the method of multimorbidity scoring. The actual EQ5D scores were moderately lower in the highest categories of multimorbidity but the relative improvement was the same. Regression analyses, both unadjusted and adjusted for age and gender showed that the post-operative scores, adjusted for the pre-operative scores, were not importantly different (see Appendix 1) for any of the multimorbidity scores compared with the lowest score in each grading system.

Discussion

Although previous studies have demonstrated a link between same indices of serious morbidity, such as the Charlson score and subsequent longer term mortality [19]; this is the first study to comprehensively assess the impact of multiple approaches to pre-operative health measures, on the broad range of risks and benefits of THA. We have demonstrated that there is a link between worse preoperative health status with risk of death and of clinically significant medical event rates, although the size and the steepness of any trend varied between the different approaches to assessing health. There is no single obvious measure which captures all the relevant dimensions. Of interest was that these approaches in general did not yield consistent differences in these results, although the number of primary care contacts was 'an outlier' in this respect and would not appear to be useful as a measure of health status.

The other important result was that THA is associated with a substantial improvement in quality of life, independent of level of pre-existing multimorbidity. Indeed, this was true for all the approaches used to assess health. This consistency in the level of benefit is thus in contrast to the variable impact between these approaches on complications of surgery.

There are some limitations of these data. Firstly, we have not considered the impact of different types of surgery and anaesthesia together with other prophylactic interventions to reduce the hazards from surgery in this group of patients. For example, the routine use of the use of thromboprophylaxis should reduce the risk of thromboembolism even in those with the highest multimorbidity. Such interventions would not, though, explain the observation that improvement in patient outcome is not materially affected by pre-exiting morbidity.

For peer review only

Secondly, inevitably in studies of this type, there is a reliance on the completeness, and quality diagnostic accuracy in the data sets available. We relied on the quality of the CPRD to provide information on the diagnosis of osteoarthritis of the hip and of the multimorbidity scores. As regards the former, a validation study using radiological and clinical information from a subsample of this cohort, described elsewhere, confirmed the diagnosis of hip osteoarthritis in 80 per cent.[20] As regards the latter there are many reports on the validity of the national datasets used for deriving multimorbidity information. [21,26]

There is also a similar reliance on the quality of the outcome data from HES. Although data from HES is widely accepted as being sufficiently valid [27] we were concerned that this data source captured a high proportion of these relevant outcome events with minimal misclassification. As a test of the quality of the outcomes data, we therefore compared our results on post-operative hazard rates with other published data to determine if such rates were broadly similar. There are limitations in such comparisons, but this analysis suggested that the observed rates of clinically significant events in the population studied did accord with those published from other countries. For example, our VTE incidence was 2.1%, published population data suggests a range of 0.7-3.9%.[28–30] Similar comparisons for MI were: present study 0.4%, literature 0.4-0.6%[31-33]; wound infection present study 1.7%, literature 2.2-2.9%.[34]

Readmission rate is also useful as an indirect measure of complications. Indeed, the impact of both being in the most frail category of eFI and number of prescriptions was similar on the rates of both readmissions (Supplementary Table 1) to that seen on complications (Table 4). The agreement was less observed with primary care contacts which reflects health seeking behaviours as well as morbidity.

Pre-operative health status had little effect on LOS, perhaps surprisingly but this may be complex to unpack given differences in anaesthetic procedure. We specifically studied multiple ways of measuring pre-existing morbidity and its impact on health, given there is no single 'best buy' and all such scores are measures of an underlying, but hard-to-measure, construct. This resulted in multiple estimates of effect. We were not attempting to prove statistical significance by finding one or more measure that was useful. More, by using multiple options, we covered as far as possible the different suggested approaches to ascertaining multimorbidity. A detailed statistical analysis identifying the concordance between these measures was outside the scope of this study, we were not attempting to pool the data to provide a single measure of health. Obviously these different measures will overlap in the patients classified. One of the strengths of this study was that the measures did not yield always similar relationships, especially with the risk studied. This is not, however, surprising as they were measuring different constructs. Despite such differences, none of the scoring systems had substantial effects on outcome. One construct of patient health that was not collected from these routine data is the patient's perception of their own status.

For peer review only - http://bmjopen.bmj.com/site/about/guidelines.xhtml

Others have shown for example that EQ-5D, measured prior to surgery, is associated with longer term mortality.[35] One problem with using such measures measured at a single point of time is that they fluctuate more than the longer interval used in this study to capture these primary-care recorded events.

There are other variables that might be confounders which have not been addressed in the analyses presented which include socio-economic status, body mass index (BMI), and cigarette smoking. We specifically did not adjust for the possible confounding effects of these variables in part because of the quality and completeness of the data. More importantly, we were concerned that the effect of these variables would most likely be mediated by their association with other comorbid disorders and interpretation of such adjusted results would be difficult.

The data on benefits were only collected at 6 months post-operatively. We cannot comment on the longer-term impact of these pre-operative measures on quality of life. This is not without challenges given the increasing likelihood over time that there may be other changes in health that will impact on overall benefit.

Finally, the patients studied in this cohort were inevitably those who underwent surgery. Our comparisons therefore were between those in the different morbidity categories for whom a decision had been made that surgery was appropriate. We could not address whether, *within each such category*, there were selective differences in surgical decision making that might be associated with the outcomes measured. One reasonable unmeasured confounder relates to prior perception of benefit. Thus, the individuals with major health issues who agree to surgery might have a higher expectation of outcome. Such individuals might then score higher on their self-reported outcomes, than otherwise similar patients who did not have surgery.

This study aimed to investigate the influence of common long-term disorders in the population and their influence on the risks and benefits from hip surgery. It is accepted that those at greatest risk from anaesthesia and major surgery should be identified, especially for an elective procedure such as THA. Pre-operative morbidity does increase the mortality risk [36-38]. By contrast, there are very little data on how the co-existing health problems affect the clinical benefits. Indeed, we have shown in a recent analysis that those THA patients in the highest American Society of Anesthesiologists (ASA)[39] grade (III and above) are at increased risk of revision surgery[40], a result that is not unexpected. ASA grades whilst routinely collected for example in national joint registers, are not extractable from primary care data systems. The underlying concern behind undertaking the current study was that, especially in older individuals, the lack of an evidence base weighing up the risks and benefits from surgery, leads to patients, their families and health care professionals, accepting that surgery should

For peer review only - http://bmjopen.bmj.com/site/about/guidelines.xhtml

be avoided. The current study suggests that the increased risks are modest and the benefits similar.

As part of the study design, we have been able to examine a number of different approaches to assessing multimorbidity in primary care patients. Indeed, that setting with the complete medical record provides the only practical one for scoring patients in clinical practice. The eFI, a recently developed tool for assessing frailty from the primary care record[25], is achieving widespread usage in the UK and elsewhere for classifying frailty in elderly patients. High eFI scores are associated with substantial increased risks of mortality, unplanned hospital admission and the need for long term care [25]. It is thus interesting that within the current dataset the differences in eFI class were, if anything more predictive of outcomes than the other approaches. It should be noted though that the proportion of the population selected for surgery in the highest eFI category was small. Further, even a high eFI class on our data, should not, of itself, be a barrier to surgery.

We conclude that in older patients requiring a hip replacement, unless they are in the small group whose anaesthetic risk precludes safe surgery, there is no evidence that their other long-term disorders should be a barrier to this life changing elective procedure.

For peer review only

Figure legend:
Flow chart of data available for analysis

Contributor Statement:
The study was conceived by AJS with SG-J and DP-A and detailed protocol produced by these authors with input from GP, KPJ, JMV and DY. AD was responsible for extracting, preparing and ensuring the quality of the datasets for analysis; analysis was undertaken by RJF supported by VS, DP-A and AJS, CW provided patient input into the design, approach to analysis and interpretation of the data. All authors contributed to the discussion of the results. The manuscript was prepared by AJS and RJF and reviewed by all authors

Competing interests:
The authors declare that they have no conflicts of interests to report

Funding
This report is independent research funded by the National Institute for Health Research (Research for Patient Benefit programme, PB-PG-0815-20024). The views expressed in this publication are those of the authors and not necessarily those of the NIHR or the Department of Health and Social Care

This study is based in part on data from the Clinical Practice Research Datalink obtained under licence from the UK Medicines and Healthcare products Regulatory Agency. The data is provided by patients and collected by the NHS as part of their care and support. The interpretation and conclusions contained in this study are those of the author/s alone
HES Data/ONS Data shown are Copyright © 2016 are re-used with the permission of The Health & Social Care Information Centre. All rights reserved.
The OPCS Classification of Interventions and Procedures, codes, terms and text is Crown copyright (2016) published by Health and Social Care Information Centre, also known as NHS Digital and licensed under the Open Government Licence available at www.nationalarchives.gov.uk/doc/open-government-licence/open-government-licence.htm.

Data sharing statement: The data that support the findings of this study are available from CPRD but restrictions apply to the availability of these data, which were used under license for the current study, and so are not publicly available. The datasets extracted from CPRD and used in the analysis during the current study are available from the corresponding author on reasonable request and consistent with the permission received from CPRD

For peer review only - http://bmjopen.bmj.com/site/about/guidelines.xhtml

References

1 NHS Digital. Provisional Quarterly Patient Reported Outcome Measures (PROMs) in England. 2017. https://digital.nhs.uk/catalogue/PUB23060.

2 National Joint Registry. National Joint Registry for England, Wales, Northern Ireland and the Isle of Man: 17th Annual Report. 2020, Table 3.H4: https://reports.njrcentre.org.uk/Portals/0/PDFdownloads/NJR%2017th%20Annual%20Report%202020.pdf.

3 Barnett K, Mercer SW, Norbury M, Watt G, Wyke S, Guthrie B. Epidemiology of multimorbidity and implications for health care, research, and medical education: a cross-sectional study. *Lancet* 2012; **380**: 37–43.

4 Violan C, Foguet-Boreu Q, Flores-Mateo G, *et al.* Prevalence, Determinants and Patterns of Multimorbidity in Primary Care: A Systematic Review of Observational Studies. *PLoS One* 2014; **9**: e102149.

5 Abad-Díez JM, Calderón-Larrañaga A, Poncel-Falcó A, *et al.* Age and gender differences in the prevalence and patterns of multimorbidity in the older population. *BMC Geriatr* 2014; **14**: 75.

6 Gale CR, Cooper C, Aihie Sayer A. Prevalence of frailty and disability: findings from the English Longitudinal Study of Ageing. Age and ageing. 2014 Oct 12;44(1):162-5.

7 Vetrano DL, Palmer K, Marengoni A, Marzetti E, Lattanzio F, Roller-Wirnsberger R, Lopez Samaniego L, Rodríguez-Mañas L, Bernabei R, Onder G, Joint Action ADVANTAGE WP4 Group. Frailty and multimorbidity: a systematic review and meta-analysis. The Journals of Gerontology: Series A. 2018 May 3;74(5):659-66.

8 Aynardi M, Jacovides CL, Huang R, Mortazavi SMJ, Parvizi J. Risk Factors for Early Mortality Following Modern Total Hip Arthroplasty. *J Arthroplasty* 2013; **28**: 517–20.

9 Rasouli MR, Restrepo C, Maltenfort MG, Purtill JJ, Parvizi J. Risk Factors for Surgical Site Infection Following Total Joint Arthroplasty. *J Bone Jt Surgery-American Vol* 2014; **96**: e158-1–5.

10 Singh JA, Jensen MR, Harmsen WS, Gabriel SE, Lewallen DG. Cardiac and thromboembolic complications and mortality in patients undergoing total hip and total knee arthroplasty. *Ann Rheum Dis* 2011; **70**: 2082–8.

11 Gordon M, Paulsen A, Overgaard S, Garellick G, Pedersen AB, Rolfson O. Factors influencing health-related quality of life after total hip replacement - a comparison of data from the Swedish and Danish hip arthroplasty registers. *BMC Musculoskelet Disord* 2013; **14**: 316.

12 Greene ME, Rolfson O, Gordon M, Garellick G, Nemes S. Standard Comorbidity Measures Do Not Predict Patient-reported Outcomes 1 Year After Total Hip Arthroplasty. *Clin Orthop Relat Res* 2015; **473**: 3370–9.

13 Loth FL, Giesinger JM, Giesinger K, *et al.* Impact of Comorbidities on Outcome After Total Hip Arthroplasty. *J Arthroplasty* 2017; **32**: 2755–61.

For peer review only - http://bmjopen.bmj.com/site/about/guidelines.xhtml

14 Glassou EN, Pedersen AB, Aalund PK, Mosegaard SB, Hansen TB: Is gain in health-related quality of life after a total hip arthroplasty depended on the comorbidity burden? Acta Orthop 2018 **89** 374-9

15 National Institue for Health and Clinical Excellence: https://www.nice.org.uk/guidance/ta304/chapter/4-Evidence-and-interpretation

16 American Academy of Orthopedic Surgeons: https://www.aaos.org/uploadedFiles/PreProduction/Quality/Guidelines_and_Reviews/OA%20Hip%20CPG_4-22-19.pdf

17 The Academy of Medical Sciences. Multimorbidity: a priority for global health research. 2018 https://acmedsci.ac.uk/file-download/82222577 (accessed April 4, 2019).

18 Charlson ME, Pompei P, Ales KL, MacKenzie CR. A new method of classifying prognostic comorbidity in longitudinal studies: development and validation. *J Chronic Dis* 1987; **40**: 373–83

19 Bulow E, Rolfson), Cnudde, *et al.* Comorbidity does not predict long-term mortality after total hip arthroplasty. *Acta Orthop* 2017; **88**; 472-77

20 Ferguson RJ, Prieto-Alhambra D, Peat G, *et al.* Influence of pre-existing multimorbidity on receiving a hip arthroplasty: Cohort study of 28,026 elderly subjects from UK primary care

21 Herrett E, Gallagher AM, Bhaskaran K, *et al.* Data Resource Profile: Clinical Practice Research Datalink (CPRD). *Int J Epidemiol* 2015; **44**: 827–36.

22 NHS Digital. Patient Reported Outcome Measures (PROMs). https://digital.nhs.uk/data-and-information/data-tools-and-services/data-services/patient-reported-outcome-measures-proms#guidance (accessed June 21, 2019).

23 Ferguson RJ, Prieto-Alhambra D, Walker C, *et al.* Validation of hip osteoarthritis diagnosis recording in the UK Clinical Practice Research Datalink. *Pharmacoepidemiol Drug Saf* 2018; **28**: 187–93.

24. NHS Primary Care Commissioning *QOF implementation business rules: v11*. http://www.primarycarecontracting.nhs.uk/145.php

25 Clegg A, Bates C, Young J, *et al.* Development and validation of an electronic frailty index using routine primary care electronic health record data. *Age Ageing* 2016; **45**: 353–60.

26 Jick SS(1), Kaye JA, Vasilakis-Scaramozza C, *et al*. Validity of the general practice research database. *Pharmacotherapy*. 2003;**23**:686-9.

27 Royal College of Surgeons of England, Academy of Medical Royal Colleges. Hospital Episode Statistics and revalidation.2013, RCS England

28 Senay A, Trottier M, Delisle J, *et al.* Incidence of symptomatic venous thromboembolism in 2372 knee and hip replacement patients after discharge: data from a thromboprophylaxis registry in Montreal, Canada. *Vasc Health Risk Manag* 2018; **Volume 14**: 81–9.

For peer review only - http://bmjopen.bmj.com/site/about/guidelines.xhtml

29 Lee S, Hwang J-I, Kim Y, Yoon PW, Ahn J, Yoo JJ. Venous Thromboembolism Following Hip and Knee Replacement Arthroplasty in Korea: A Nationwide Study Based on Claims Registry. *J Korean Med Sci* 2016; **31**: 80.

30 Pedersen AB, Sorensen HT, Mehnert F, Overgaard S, Johnsen SP. Risk Factors for Venous Thromboembolism in Patients Undergoing Total Hip Replacement and Receiving Routine Thromboprophylaxis. *J Bone Jt Surgery-American Vol* 2010; **92**: 2156–64.

31 Wallace G, Judge A, Prieto-Alhambra D, de Vries F, Arden NKNK, Cooper C. The effect of body mass index on the risk of post-operative complications during the 6 months following total hip replacement or total knee replacement surgery. *Osteoarthr Cartil* 2014; **22**: 918–27.

32 Pedersen AB, Mehnert F, Sorensen HT, Emmeluth C, Overgaard S, Johnsen SP. The risk of venous thromboembolism, myocardial infarction, stroke, major bleeding and death in patients undergoing total hip and knee replacement. *Bone Joint J* 2014; **96-B**: 479–85.

33 Belmont PJ, Goodman GP, Kusnezov NA, *et al.* Postoperative Myocardial Infarction and Cardiac Arrest Following Primary Total Knee and Hip Arthroplasty: Rates, Risk Factors, and Time of Occurrence. *J Bone Jt Surgery-American Vol* 2014; **96**: 2025–31

34 Friedman R, Homering M, Holberg G, Berkowitz SD. Allogeneic Blood Transfusions and Postoperative Infections After Total Hip or Knee Arthroplasty. *J Bone Jt Surg* 2014; **96**: 272–8.

35 Cnudde P, Nemes S, Mohaddes M, *et al*. Is pre-operative patient-reported health status associated with mortality after total hip replacement? Int J Environ Pub Health Res 2017; **14** 899-907.

36 Aynardi M, Jacovides CL, Huang R, Mortazavi SMJ, Parvizi J. Risk Factors for Early Mortality Following Modern Total Hip Arthroplasty. *J Arthroplasty* 2013; **28**: 517–20

37 Illingworth KD, El Bitar YF, Banerjee D, Scaife SL, Saleh KJ. Inpatient Mortality After Primary Total Hip Arthroplasty: Analysis from the National Inpatient Sample Database. *J Arthroplasty* 2015; **30**: 369–73.

38 Singh JA, Jensen MR, Harmsen WS, Gabriel SE, Lewallen DG. Cardiac and thromboembolic complications and mortality in patients undergoing total hip and total knee arthroplasty. *Ann Rheum Dis* 2011; **70**: 2082–8.

39 Doyle JD, Garmon EH. American Society of Anesthesiologists Classification (ASA Class) https://www.ncbi.nlm.nih.gov/books/NBK441940/

40 Ferguson RJ, Silman AJ, Combescure C), *et al.* ASA class is associated with early revision and reoperation after total hip arthroplasty: an analysis of the Geneva and Swedish Hip Arthroplasty Registries. Acta Orthop. 2019;**90**:324-330.

For peer review only

Table 1: Demographic characteristics of cohort

**Gender**	N (%)
Female	4,090 (61.2)
**Age**	
65-69	1,294 (19.4)
70-74	1,976 (29.6)
75-79	1,736 (26.0)
80-84	1,095 (16.4)
85-89	465 (7.0)
>90	116 (1.7)
**Body Mass Index (BMI)**	
Underweight (<18.5)	54 (1.0)
Normal: (18.5-24.9)	1,615 (28.8)
Overweight: (25-29.9)	2,444 (43.6)
Obese: (>30)	1,497 (26.7)
Missing	1072
**Index of multiple deprivation**	
1 (Affluent)	1076 (25.5)
2	1718 (25.7)
3	1539 (23.0)
4	1144 (17.1)
5 (Deprived)	573 (8.6)
Missing	2

For peer review only

Table 2: Baseline data on measures of pre-operative health status (multimorbidity, frailty and morbidity burden)

**Charlson comorbidity index**	
0	4,490 (67.2)
1	682 (10.2)
2	880 (13.2)
3+	630 (9.4)
**Electronic frailty index (eFI)**	
0-4	4184 (62.6)
5-8	2,064 (30.9)
9-12	393 (5.9)
13+	41 (0.6)
**No. comorbid diseases**	
0	1,901 (28.5)
1	2,286 (34.2)
2	1,430 (21.4)
3+	1,065 (15.9)
**No. medications prescribed**	
0-4	1,963 (29.4)
5-7	1,832 (27.4)
8-12	1,992 (29.8)
13+	895 (13.4)
**No. contacts with primary care**	
0-7	2,167 (32.4)
8-11	1,598 (23.9)
12-17	1,528 (22.9)
18+	1,389 (20.8)

For peer review only

Table 3: Complications, length of stay, and readmission to hospital within 90 days

	N (%)
Any medical complication*	216 (3.2)
Death	57 (0.9)
Myocardial infarction	20 (0.3)
Venous thrombo-embolism	54 (0.8)
Stroke	8 (0.1)
Anaemia	12 (0.2)
Respiratory tract infection	28 (0.4)
Urinary tract infection	12 (0.2)
Wound infection	37 (0.6)
Length of stay (median, IQR)	6 (4-9)
Readmission to hospital	727 (10.9)

*Any medical complication includes death, myocardial infarction, venous thrombo-embolism, stroke, anaemia, respiratory tract infection, urinary tract infection, and wound infection

For peer review only

For peer review only - http://bmjopen.bmj.com/site/about/guidelines.xhtml

Table 4: Association between pre-operative morbidity status and 90-day medical complications

	No. at risk	Events	% Events	Unadjusted OR	Adjusted OR*
Charlson CI					
0	4490	120	2.67	Ref	Ref
1	682	27	3.96	1.50 (0.98, 2.30)	1.43 (0.93, 2.20)
2	880	43	4.89	1.87 (1.31, 2.67)	1.78 (1.24, 2.56)
3+	630	26	4.13	1.57 (1.02, 2.42)	1.46 (0.94, 2.26)
EFI					
0-4	4184	112	2.68	Ref	Ref
5-8	2064	75	3.63	1.37 (1.02, 1.85)	1.31 (0.96, 1.78)
9-12	393	26	6.62	2.58 (1.66, 4.00)	2.26 (1.42, 3.62)
13+	41	<5**			
Comorbid diseases					
0	1901	53	2.79	Ref	Ref
1	2286	53	2.32	0.83 (0.56, 1.22)	0.80 (0.54, 1.18)
2	1430	57	3.99	1.45 (0.99, 2.12)	1.41 (0.96, 2.08)
3+	1065	53	4.98	1.83 (1.24, 2.69)	1.74 (1.16, 2.61)
Prescriptions					
0-4	1963	49	3.09	Ref	Ref
5-7	1832	55	2.75	1.21 (0.82, 1.79)	1.17 (0.79, 1.73)
8-12	1992	68	3.53	1.38 (0.95, 2.00)	1.28 (0.88, 1.87)
13+	895	33	3.67	2.02 (1.33, 3.06)	1.85 (1.21, 2.83)
PC contacts					
0-7	2167	67	3.09	Ref	Ref
8-11	1598	44	2.75	0.89 (0.60, 1.31)	0.85 (0.58, 1.25)
12-17	1528	54	3.53	1.15 (0.80, 1.65)	1.09 (0.76, 1.58)
18+	1389	51	3.67	1.19 (0.82, 1.73)	1.07 (0.74, 1.57)
Total	6682	216			

*Adjusted for age, gender, region and calendar year of surgery

** The rules set for approval of use of CPRD data require that authors are not allowed to indicate the number of events in any category if they are less than 5

For peer review only - http://bmjopen.bmj.com/site/about/guidelines.xhtml

1 **Table 5: Association between pre-operative multimorbidity and 90-day mortality**

2

	No. at risk	Events	% Events	Unadjusted OR	Adjusted OR*
Charlson CI					
0	4490	32	0.71	Ref	Ref
1	682	6	0.88	1.24 (0.52, 2.97)	1.09 (0.45, 2.65)
2	880	13	1.48	2.09 (1.09, 4.00)	1.85 (0.95, 3.60)
3+	630	6	0.95	1.34 (0.56, 3.22)	1.12 (0.46, 2.73)
EFI					
0-4	4184	27	0.65	Ref	Ref
5-8	2064	20	0.97	1.51 (0.84, 2.69)	1.33 (0.72, 2.44)
9-12	393	10	2.54	4.02 (1.93, 8.37)	2.78 (1.24, 6.23)
13+	41	0	0	-	-
Comorbid diseases					
0	1901	14	0.74	Ref	Ref
1	2286	10	0.44	0.59 (0.26, 1.34)	0.58 (0.26, 1.33)
2	1430	16	1.12	1.53 (0.74, 3.14)	1.50 (0.71, 3.16)
3+	1065	17	1.60	2.19 (1.07, 4.45)	2.12 (0.99, 4.51)
Prescriptions					
0-4	1963	11	0.56	Ref	Ref
5-7	1832	19	1.04	1.86 (0.88, 3.92)	1.80 (0.85, 3.82)
8-12	1992	18	0.90	1.62 (0.76, 3.43)	1.39 (0.64, 2.98)
13+	895	9	1.01	1.80 (0.74, 4.37)	1.51 (0.61, 3.75)
Primary Care contacts					
0-7	2167	20	0.92	Ref	Ref
8-11	1598	13	0.81	0.88 (0.44, 1.78)	0.81 (0.40, 1.65)
12-17	1528	15	0.98	1.06 (0.54, 2.09)	0.90 (0.45, 1.78)
18+	1389	9	0.65	0.70 (0.32, 1.54)	0.53 (0.24, 1.19)
Total	6682	57			

3

4 *Adjusted for age, gender, region and calendar year of surgery

For peer review only

Table 6: Patient Reported Outcome Measures (PROMs)

	N	Mean (SD)
Oxford Hip Score		
Pre-operative	1460	17.9 (7.9)
Post-operative	1416	39.3 (8.2)
Change	1402	21.3 (9.7)
EuroQoL Index (EQ-5D)		
Pre-operative	1380	0.35 (0.31)
Post-operative	1367	0.80 (0.23)
Change	1285	0.45 (34)

For peer review only

Table 7: Post-operative Oxford Hip Score by multimorbidity

	No.	Pre-op	Post-op	Change	Unadjusted difference	Adjusted*
Charlson CI						
0	960	18.5 (8.0)	39.9 (8.0)	21.4 (9.6)	Ref	Ref
1	113	17.6 (8.4)	37.5 (9.7)	20.1 (10.5)	-2.0 (-3.6, -0.5)	-2.0 (-3.6, -0.5)
2	199	16.0 (7.5)	37.8 (8.3)	21.9 (9.4)	-1.4 (-2.6, -0.2)	-1.0 (-2.2, 0.2)
3+	130	16.8 (7.1)	38.5 (8.1)	21.5 (10.0)	-1.15 (-2.5, 0.4)	-0.60 (-2.0, 0.8)
EFI						
0-4	798	18.8 (7.9)	40.7 (7.5)	21.8 (9.4)	Ref	Ref
5-8	511	17.3 (7.9)	37.7 (8.8)	20.5 (10.2)	-2.5 (-3.4, -1.7)	-2.0 (-2.9, -1.2)
9-12	86	14.6 (7.0)	35.8 (8.1)	21.4 (8.8)	-3.6 (-5.4, -1.9)	-2.9 (-4.7, -1.1)
13+	7	14.7 (8.2)	39.1 (10.4)	24.4 (8.7)	-0.45 (-6.2, 5.3)	1.0 (-4.8, 6.7)
Comorbid dis						
0	358	18.9 (7.8)	40.7 (7.9)	21.7 (9.9)	Ref	Ref
1	481	18.6 (8.1)	39.9 (7.9)	21.3 (9.6)	-0.7 (-1.8, 0.4)	-0.4 (-1.5, 0.6)
2	324	17.6 (7.9)	38.7 (8.2)	21.0 (9.6)	-1.6 (-2.8 , -0.4)	-1.2 (-2.3, 0.1)
3+	239	15.7 (7.5)	36.9 (8.9)	21.2 (9.7)	-3.0 (-4.3, -1.7)	-2.3 (-3.6, -1.0)
Prescriptions						
0-4	407	19.9 (7.4)	41.4 (7.1)	21.4 (9.3)	Ref	Ref
5-7	383	19.4 (8.1)	40.4 (7.4)	20.9 (9.5)	-0.8 (-1.9, 0.3)	-0.5 (-1.6, 0.6)
8-12	406	16.6 (7.7)	38.2 (8.5)	21.7 (9.9)	-2.3 (-3.4, -1.2)	-2.0 (-3.0, -0.9)
13+	206	14.1 (7.3)	35.4 (9.2)	21.3 (10.3)	-4.6 (-5.9, -3.2)	-4.0 (-5.3, -2.4)
PC contacts						
0-7	2167	18.9 (7.8)	39.9 (7.8)	21.0 (8.9)	Ref	Ref
8-11	1598	18.3 (8.1)	40.1 (7.8)	21.7 (10.0)	0.3 (-0.8 , 1.5)	0.6 (-0.6, 1.7)
12-17	1528	17.8 (7.8)	39.4 (8.1)	21.7 (9.9)	-0.1 (-1.2, 1.0)	0.4 (-0.7, 1.5)
18+	1389	16.1 (9.1)	37.2 (9.2)	21.1 (10.3)	-2.0 (-3.2, -0.8)	-1.5 (-2.7, -0.3)
Total	1402	1460	1416	1402		

*Adjusted for age, gender, region and calendar year of surgery

For peer review only

For peer review only - http://bmjopen.bmj.com/site/about/guidelines.xhtml

Table 8: Post-operative EQ5D by multimorbidity

	No.	Pre-op	Post-op	Change	Unadjusted difference	Adjusted difference*
Charlson CI						
0	880	.37 (.31)	.82 (.22)	.45 (.34)	Ref	Ref
1	108	.33 (.32)	.77 (.25)	.44 (.33)	-.036 (-.080, .007)	-.037 (-.080, .007)
2	178	.29 (.31)	.76 (.22)	.48 (.34)	-.038 (-.073, -.002)	-.028 (-.064, .007)
3+	119	.33 (.29)	.74 (.24)	.40 (.33)	-.072 (-.114, -.030)	-.060 (-.102, -.018)
EFI						
0-4	735	.39 (.31)	.83 (.20)	.45 (.33)	Ref	Ref
5-8	464	.32 (.31)	.76 (.23)	.45 (.36)	-.059 (-.084, -.033)	-.048 (-.073, -.022)
9-12	79	.20 (.29)	.69 (.28)	.47 (.34)	-.141 (-.192, -.090)	-.126 (-.178, -.074)
13+	7	.20 (.35)	.78 (.35)	.59 (.32)	-.021 (-.184, .141)	.004 (-.157, .166)
Comorbid dis						
0	330	.39 (.31)	.85 (.19)	.46 (.34)	Ref	Ref
1	429	.38 (.31)	.81 (.20)	.43 (.32)	-.037 (-.068, -.005)	-.030 (-.062, .001)
2	306	.33 (.31)	.78 (.23)	.45 (.35)	-.060 (-.094, -.026)	-.049 (-.084, -.015)
3+	220	.26 (.32)	.71 (.28)	.45 (.36)	-.122 (-.159, -.084)	-.105 (-.143, -.067)
Prescriptions						
0-4	378	.42 (.30)	.85 (.20)	.44 (.34)	Ref	Ref
5-7	351	.41 (.30)	.83 (.20)	.42 (.32)	-.023 (-.055, .008)	-.062 (-.062, .001)
8-12	372	.30 (.30)	.77 (.22)	.47 (.34)	-.071 (-.103, -.040)	-.084 (-.084, -.015)
13+	184	.19 (.31)	.69 (.28)	.50 (.37)	-.139 (-.178, -.099)	-.143 (-.143, -.067)
PC contacts						
0-7	2167	.39 (.31)	.82 (.22)	.44 (.33)	Ref	Ref
8-11	1598	.36 (.30)	.83 (.21)	.46 (.34)	.003 (-.029, .036)	.007 (-.025, .039)
12-17	1528	.36 (.31)	.80 (.21)	.44 (.35)	-.018 (-.049, .013)	-.010 (-.042, .021)
18+	1389	.26 (.32)	.72 (.26)	.47 (.35)	-.079 (-.114, -.044)	-.070 (-.106, -.034)
Total	1285	1380	1367	1285		

*Adjusted for age, gender, region and calendar year of surgery

For peer review only

Figure: Flow chart of available data

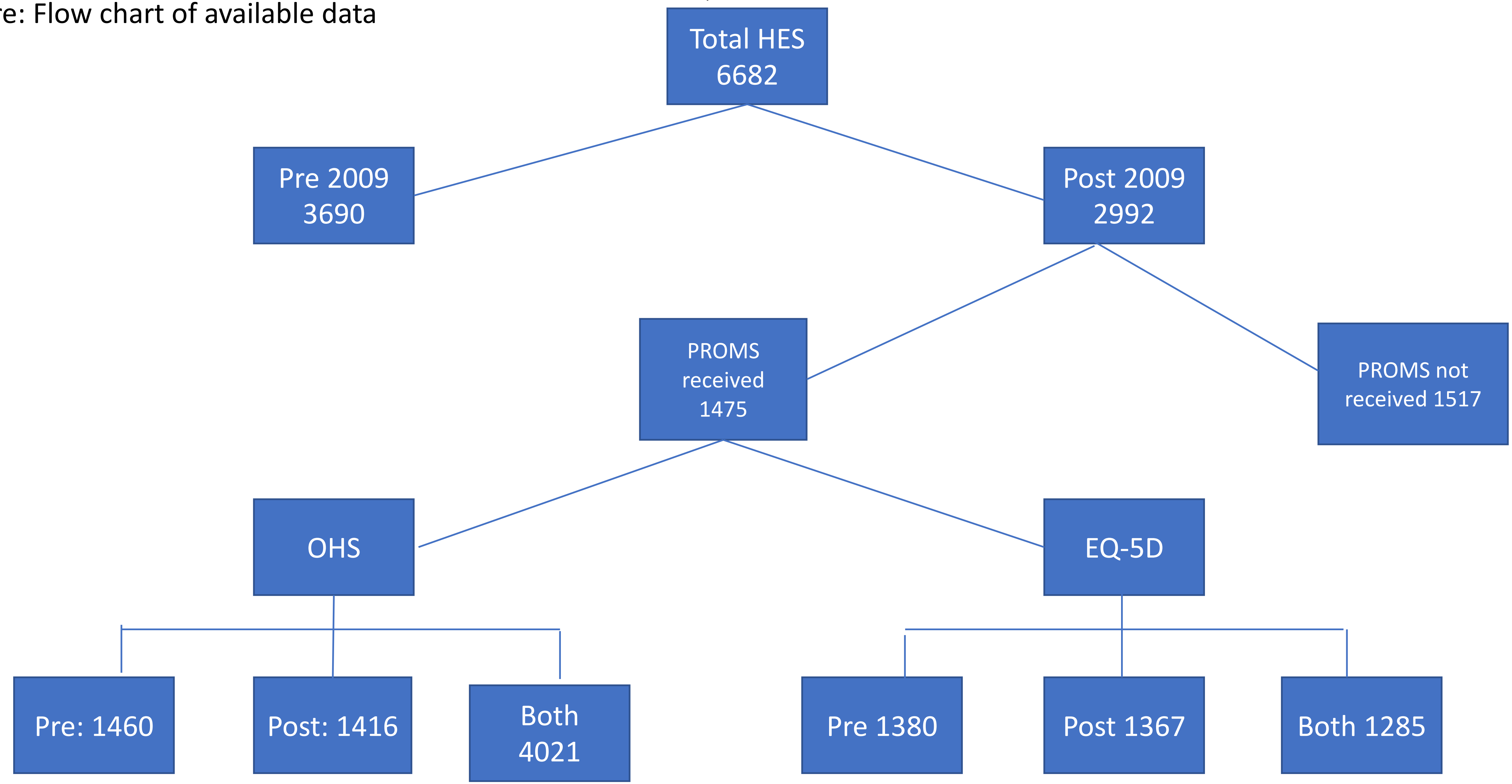

Appendix: Interpretation of Oxford Hip Scores and EQ-5D in patients receiving a hip arthroplasty

The OHS is a 12-item score with 4 levels, thus scores vary between 0-48, with higher scores being the best outcome. The clinically meaningful difference is considered to be an increase of +11 points and a change of +5 is considered statistically meaningful (ie greater than background 'noise')

Beard DJ, Harris K, Dawson J, *et al.* Meaningful changes for the Oxford hip and knee scores after joint replacement surgery. *J Clin Epidemiol* 2015; **68**: 73–9.

The EQ-5D has been very widely studied in several disease areas. There have been several studies looking at what is a clinically meaningful difference and a meta-analysis yielded an average difference of 0.18

Coretti S, Ruggeri M, McNamee P. The minimum clinically important difference for EQ-5D index: A critical review. Expert Rev. Pharmacoeconomics Outcomes Res. 2014; **14**: 221–33

For peer review only

Supplementary Table 1: Baseline data on measures of pre-operative health status (multimorbidity, frailty and morbidity burden) in patients who underwent primary THR in HES APC during the date period in which PROMs were collected (N=2992)

	PROMs data	No PROMs data
**Charlson comorbidity index**		
0	1,013 (68.7)	931 (61.4)
1	123 (8.3)	152 (10.0)
2	205 (13.9)	254 (16.7)
3+	134 (9.1)	180 (11.9)
**Electronic frailty index (eFI)**		
0-4	831 (56.3)	752 (49.6)
5-8	544 (36.9)	593 (39.1)
9-12	93 (6.3)	151 (10.0)
13+	7 (0.5)	21 (1.4)
**No. comorbid diseases**		
0	378 (25.6)	320 (21.1)
1	497 (33.7)	464 (30.6)
2	346 (23.5)	364 (24.0)
3+	254 (17.2)	369 (24.3)
**No. medications prescribed**		
0-4	428 (29.0)	400 (26.4)
5-7	401 (27.2)	375 (24.7)
8-12	429 (29.1)	500 (33.0)
13+	217 (14.7)	242 (16.0)
**No. contacts with primary care**		
0-7	471 (31.9)	464 (30.6)
8-11	335 (22.7)	358 (23.6)
12-17	384 (26.0)	334 (22.0)
18+	285 (19.3)	361 (23.8)
**Total**	1475	1517

For peer review only

Supplementary Table 2

Influence of pre-operative health status on risk of hospital readmission within 90 days

	No. patients	Events	% Events	Unadjusted OR (95% CI)	Adjusted OR* (95% CI)
Charlson Comorbidity Index					
0	4490	477	10.6	Ref	Ref
1	682	82	12.0	1.15 (0.90, 1.48)	1.09 (0.85, 1.40)
2	880	131	14.9	1.47 (1.19, 1.81)	1.41 (1.14, 1.74)
3+	630	96	15.2	1.51 (1.19, 1.92)	1.45 (1.14, 1.84)
Count of chronic diseases					
0	1901	192	10.1	Ref	Ref
1	2286	232	10.2	1.01 (0.82, 1.23)	1.01 (0.82, 1.23)
2	1430	205	14.3	1.49 (1.21, 1.84)	1.47 (1.19, 1.83)
3+	1065	157	14.7	1.54 (1.23, 1.93)	1.53 (1.21, 1.94)
Count of medications prescribed					
0-4	1963	180	9.2	Ref	Ref
5-7	1832	201	11.0	1.22 (0.99, 1.51)	1.22 (0.99, 1.51)
8-12	1992	257	12.9	1.47 (1.20, 1.80)	1.45 (1.18, 1.78)
13+	895	148	16.5	1.96 (1.55, 2.48)	1.96 (1.54, 2.49)
Count of contacts with primary care					
0-7	2167	204	9.4	Ref	Ref
8-11	1598	172	10.8	1.16 (0.94, 1.44)	1.15 (0.93, 1.43)
12-17	1528	192	12.6	1.38 (1.12, 1.70)	1.37 (1.11, 1.69)
18+	1389	218	15.7	1.79 (1.46, 2.20)	1.75 (1.42, 2.15)
Electronic Frailty Index					
0-4	4184	432	10.3	Ref	Ref
5-8	2064	270	13.1	1.31 (1.11, 1.54)	1.30 (1.10, 1.55)
9-12	393	76	19.3	2.08 (1.59, 2.73)	2.01 (1.51, 2.67)
13+	41	8	19.5	2.11 (0.97, 4.59)	2.15 (0.97, 4.73)
Total	6682	786	11.8		

*The model was adjusted for age, sex, region of the residence, and year of surgery.

For peer review only

Supplementary Table 3:

Influence of pre-operative health status on length of stay in hospital

	Total at risk	Median length of stay (IQR)
Charlson Comorbidity Index		
0	4490	6 (4, 9)
1	682	6 (5, 9)
2	880	6 (4, 9)
3+	630	7 (5, 9)
Count of chronic diseases		
0	1901	6 (4, 8)
1	2286	6 (4, 9)
2	1430	6 (4, 9)
3+	1065	7 (5, 10)
Count of medications prescribed		
0-4	1963	6 (4, 8)
5-7	1832	6 (4, 9)
8-12	1992	7 (5, 9)
13+	895	7 (5, 10)
Count of contacts with primary care		
0-7	2167	6 (4, 8)
8-11	1598	6 (4, 9)
12-17	1528	6 (4, 9)
18+	1389	7 (5, 10)
Electronic Frailty Index		
0-4	4184	6 (4, 9)
5-8	2064	6 (4, 9)
9-12	393	8 (5, 11)
13+	41	8 (7, 13)
Total	6682	6 (4, 9)

| ,

For peer review only

For peer review only

Reporting checklist for cohort study.

Based on the STROBE cohort guidelines.

Instructions to authors

Complete this checklist by entering the page numbers from your manuscript where readers will find each of the items listed below.

Your article may not currently address all the items on the checklist. Please modify your text to include the missing information. If you are certain that an item does not apply, please write "n/a" and provide a short explanation.

Upload your completed checklist as an extra file when you submit to a journal.

In your methods section, say that you used the STROBE cohortreporting guidelines, and cite them as:

von Elm E, Altman DG, Egger M, Pocock SJ, Gotzsche PC, Vandenbroucke JP. The Strengthening the Reporting of Observational Studies in Epidemiology (STROBE) Statement: guidelines for reporting observational studies.

		Reporting Item	Page Number (lines)
**Title and abstract**			
Title	#1a	Indicate the study's design with a commonly used term in the title or the abstract	1
Abstract	#1b	Provide in the abstract an informative and balanced summary of what was done and what was found	3
**Introduction**			
Background / rationale	#2	Explain the scientific background and rationale for the investigation being reported	5
Objectives	#3	State specific objectives, including any prespecified hypotheses	5 (22-25)
**Methods**			
Study design	#4	Present key elements of study design early in the paper	5 (34-37)

For peer review only

Setting	#5	Describe the setting, locations, and relevant dates, including periods of recruitment, exposure, follow-up, and data collection	6 (5-14)
Eligibility criteria	#6a	Give the eligibility criteria, and the sources and methods of selection of participants. Describe methods of follow-up.	6 (17-22)
Eligibility criteria	#6b	For matched studies, give matching criteria and number of exposed and unexposed	NA
Variables	#7	Clearly define all outcomes, exposures, predictors, potential confounders, and effect modifiers. Give diagnostic criteria, if applicable	6 (24)-7 (3)
Data sources / measurement	#8	For each variable of interest give sources of data and details of methods of assessment (measurement). Describe comparability of assessment methods if there is more than one group. Give information separately for for exposed and unexposed groups if applicable.	6 (17) – 7 (3)
Bias	#9	Describe any efforts to address potential sources of bias	8 (5-9)
Study size	#10	Explain how the study size was arrived at	7 (27-30)
Quantitative variables	#11	Explain how quantitative variables were handled in the analyses. If applicable, describe which groupings were chosen, and why	7 (6-14)
Statistical methods	#12a	Describe all statistical methods, including those used to control for confounding	7 (17-22)
Statistical methods	#12b	Describe any methods used to examine subgroups and interactions	NA
Statistical methods	#12c	Explain how missing data were addressed	8 (1-9)
Statistical methods	#12d	If applicable, explain how loss to follow-up was addressed	NA
Statistical methods	#12e	Describe any sensitivity analyses	8 (5-9)
**Results**			
Participants	#13a	Report numbers of individuals at each stage of study—eg numbers potentially eligible, examined for eligibility, confirmed eligible,	Figure

For peer review only

		included in the study, completing follow-up, and analysed. Give information separately for for exposed and unexposed groups if applicable.	
Participants	#13b	Give reasons for non-participation at each stage	NA
Participants	#13c	Consider use of a flow diagram	Figure
Descriptive data	#14a	Give characteristics of study participants (eg demographic, clinical, social) and information on exposures and potential confounders. Give information separately for exposed and unexposed groups if applicable.	Table 1
Descriptive data	#14b	Indicate number of participants with missing data for each variable of interest	Table 1
Descriptive data	#14c	Summarise follow-up time (eg, average and total amount)	6 (35-41)
Outcome data	#15	Report numbers of outcome events or summary measures over time. Give information separately for exposed and unexposed groups if applicable.	Tables 3,6
Main results	#16a	Give unadjusted estimates and, if applicable, confounder-adjusted estimates and their precision (eg, 95% confidence interval). Make clear which confounders were adjusted for and why they were included	Tables 4,5,7,8
Main results	#16b	Report category boundaries when continuous variables were categorized	Table 2
Main results	#16c	If relevant, consider translating estimates of relative risk into absolute risk for a meaningful time period	NA
Other analyses	#17	Report other analyses done—e.g., analyses of subgroups and interactions, and sensitivity analyses	Supp Table 1
**Discussion**			
Key results	#18	Summarise key results with reference to study objectives	9
Limitations	#19	Discuss limitations of the study, taking into account sources of potential bias or imprecision. Discuss both direction and magnitude of any potential bias.	9-11

For peer review only

Interpretation	#20	Give a cautious overall interpretation considering objectives, limitations, multiplicity of analyses, results from similar studies, and other relevant evidence.	11
Generalisability	#21	Discuss the generalisability (external validity) of the study results	11
**Other Information**			
Funding	#22	Give the source of funding and the role of the funders for the present study and, if applicable, for the original study on which the present article is based	2

The STROBE checklist is distributed under the terms of the Creative Commons Attribution License CC-BY. This checklist was completed on 20. October 2020 using https://www.goodreports.org/, a tool made by the EQUATOR Network in collaboration with Penelope.ai

For peer review only
